# High-Performance Drug Discovery: Computational Screening by Combining Docking and Molecular Dynamics Simulations

**DOI:** 10.1371/journal.pcbi.1000528

**Published:** 2009-10-09

**Authors:** Noriaki Okimoto, Noriyuki Futatsugi, Hideyoshi Fuji, Atsushi Suenaga, Gentaro Morimoto, Ryoko Yanai, Yousuke Ohno, Tetsu Narumi, Makoto Taiji

**Affiliations:** 1 High-performance Molecular Simulation Team, Computational Systems Biology Research Group, Advanced Computational Sciences Department, RIKEN Advanced Science Institute, Yokohama, Kanagawa, Japan; 2 High-performance Computing Team, Integrated Simulation of Living Matter Group, Computational Science Research Program, RIKEN, Yokohama, Kanagawa, Japan; 3 Graduate School of Pharmaceutical Sciences, Chiba University, Chiba, Japan; 4 Department of Computer Science, The University of Electro-Communications, Chofu-shi, Tokyo, Japan; Rutgers University, United States of America

## Abstract

Virtual compound screening using molecular docking is widely used in the discovery of new lead compounds for drug design. However, this method is not completely reliable and therefore unsatisfactory. In this study, we used massive molecular dynamics simulations of protein-ligand conformations obtained by molecular docking in order to improve the enrichment performance of molecular docking. Our screening approach employed the molecular mechanics/Poisson-Boltzmann and surface area method to estimate the binding free energies. For the top-ranking 1,000 compounds obtained by docking to a target protein, approximately 6,000 molecular dynamics simulations were performed using multiple docking poses in about a week. As a result, the enrichment performance of the top 100 compounds by our approach was improved by 1.6–4.0 times that of the enrichment performance of molecular dockings. This result indicates that the application of molecular dynamics simulations to virtual screening for lead discovery is both effective and practical. However, further optimization of the computational protocols is required for screening various target proteins.

## Author Summary

Lead discovery is one of the most important processes in rational drug design. To improve the rate of the detection of lead compounds, various technologies such as high-throughput screening and combinatorial chemistry have been introduced into the pharmaceutical industry. However, since these technologies alone may not improve lead productivity, computational screening has become important. A central method for computational screening is molecular docking. This method generally docks many flexible ligands to a rigid protein and predicts the binding affinity for each ligand in a practical time. However, its ability to detect lead compounds is less reliable. In contrast, molecular dynamics simulations can treat both proteins and ligands in a flexible manner, directly estimate the effect of explicit water molecules, and provide more accurate binding affinity, although their computational costs and times are significantly greater than those of molecular docking. Therefore, we developed a special purpose computer “MDGRAPE-3” for molecular dynamics simulations and applied it to computational screening. In this paper, we report an effective method for computational screening; this method is a combination of molecular docking and massive-scale molecular dynamics simulations. The proposed method showed a higher and more stable enrichment performance than the molecular docking method used alone.

## Introduction

In early-phase drug development research, new lead compounds are detected by the computational screening of large compound libraries. Since the goal of computational screening is basically the same as that of experimental screening, i.e., high-throughput screening (HTS), it is expected that the integration and improvement of computational and experimental approaches will increase the productivity of drug discovery. HTS is currently widely adopted and is crucial to the generation of lead compounds. Despite the many successes achieved with HTS [1]–[5], there remain some problems regarding the cost, complexity of the assay procedure, and screening quality [5]–[8]. Thus, HTS alone may not improve lead productivity. Hence, computational screening methods, such as ligand- and structure-based screening, have become important. With the advancement of computer performance and calculation techniques, computational screening has become faster and less expensive than HTS. However, the ability of computational screening to enrich hit compounds remains unsatisfactory and less reliable.

Coupled with a rapidly rising number of structures for target proteins, structure-based screening has become prominent in drug discovery. Among the various structure-based computational methodologies adopted for compound screening, the principal one is molecular docking. When the three-dimensional structure of a target protein is available or can be modeled, molecular docking is often used for the screening of compound libraries. Molecular docking predicts the conformation of a protein-ligand complex and calculates the binding affinity. Most docking programs [9]–[15] involve two operations: “docking” and “scoring.” The first involves the generation of multiple protein-ligand conformations, called “poses,” or the sampling of the ligand's probable conformations in the binding pocket of the target protein. Most of these programs perform flexible ligand-rigid receptor docking, and some of them are highly capable of predicting poses that resemble the experimental structure for many target proteins [16]. Since such docking programs enable a fast conformational search of ligands in a short time, they are very attractive tools for compound screening. In the second operation, the affinity between the target protein and the ligand for each pose is calculated by using a scoring function. Then, multiple ligands are ranked according to these calculated binding affinities or docking scores. Many studies using docking programs have shown that these screenings have a higher enrichment of hits than random screening [17],[18], but these screenings suffer from false positives and false negatives and are not sufficiently accurate to grade compounds according to the binding affinities [19]. This implies that the compounds with a higher rank include false positives and false negatives; thus, there is a practical difficulty with using docking. The problems of molecular docking as a screening tool have been widely discussed: the scoring functions are inaccurate and neglect the solvent-related terms, and protein flexibility is ignored. Furthermore, the docking score corresponding to binding free energy is less reliable because it is calculated using a single conformation even though the binding free energy is an ensemble property.

Molecular dynamics (MD) simulations can treat both proteins and ligands in a flexible manner, allowing the relaxation of the binding site around the ligand. In addition, they can directly estimate the effect of explicit water molecules. Further, more accurate MD-based computational techniques are available for estimating the binding free energy. These techniques include thermodynamic integration (TI) [20], free energy perturbation (FEP) [20], linear interaction energy (LIE) [21], and molecular mechanics/Poisson-Boltzmann and surface area (MM/PB-SA) [22] methods. The most rigorous computational techniques are the TI and FEP methods, but these are too expensive to be employed in computational screening. The computational cost of LIE is moderate, but it requires information regarding the binding affinities of experimentally known compounds. Hence, we focused on the MM/PB-SA method because many recent investigations have revealed that this method is highly capable of predicting the binding free energy [23]; further, its computational cost is lower than the computational costs of the FEP and TI methods by at least 10-fold, and its broad applicability is suitable for compound screening. In the MM/PB-SA method, the free energy is calculated using the snapshots of solute molecules obtained from explicit-solvent MD simulation. At this time, the explicit-solvent is replaced with implicit models (see Materials and Methods). These MD-based techniques can provide more accurate binding free energy, but their computational costs are considerably high, as compared to molecular docking. Further, the prediction of the optimal structures for protein-ligand complexes adds to the computational cost, even with extended-ensemble MD methods.

To solve the problem of molecular docking and MD simulations, a combination of molecular docking and MD simulations is effective because it can neutralize each other's defects. However, since the application of the MD technique to screening requires the execution of many MD simulations, the problem of the high computational cost of MD simulations remains unresolved. Because of this problem, most of the studies that have used MD-based computational techniques have reported only their ability to rank several ligands according to their experimental binding affinities [23],[24]. Further, since the most important parameter for screening is the ability to distinguish true active compounds from a large number of inactive compounds, only a few researches have assessed the ability to enrich active compounds by virtual screening using MD-based computational techniques [25],[26].

In order to reduce the significant computational cost of MD simulations, we used a special-purpose computer for MD simulations, “MDGRAPE-3,” which functions with a high speed and accuracy [27],[28]. In this study, we performed MD simulations of multiple protein-ligand conformations (multiple poses) rather than a single protein-ligand conformation (single pose). The multiple protein-ligand conformations were obtained from the result of molecular docking. Multiple poses were used so that the multiple local energy minima in the ligand's conformational space within the binding pocket could be sampled in the initial structures for MD simulations. Then, we performed massive MD simulations using multiple poses in a practically appropriate time for drug discovery.

In our screening approach, we adopted molecular docking and the MM/PB-SA method as the first and second filters for compound screening; this idea was inspired by the approach adopted by Kuhn and coworkers [25]. They made some important discoveries with respect to MD-based screening for lead generation. Their results showed that the application of the MM/PB-SA method to an energy-minimized complex structure is an adequate and more accurate approach than the calculation of the binding free energy using MD simulation. This is because the use of MD simulations introduces additional structural uncertainties and the free energy from the MD simulations leads to inaccuracy. Further, they reported that the strategy of using multiple poses cannot be recommended in general, and is useful only if the correct binding mode is contained within the higher-scored docking conformations but is not captured with a single pose. Their MD simulations were applied to the protein-ligand complexes for the top 200 compounds obtained by molecular docking, and the MD run for each complex was performed for 200 ps (with a time step of 1.5 fs). They concluded that a more sophisticated MD procedure involving an extended simulation time improved the results, although this time-consuming approach would not be of considerable interest as a tool for lead discovery.

In our study, we attempted to investigate whether a combination of molecular docking and massive-scale MD simulations would be effective in screening compound libraries. Furthermore, we evaluated which protocols for the MM/PB-SA method were effective for compound screening. In the basic MD strategy for our compound screening, a 700-ps MD simulation (with a time step of 0.5 fs) for each complex was performed for the top 1,000 compounds obtained by docking. With regard to the time resolution, simulation time, and number of protein-ligand complex structures, our MD runs were more massive and elaborate than those of previous MD-based screenings [25].

### Overview of Our Approach

In our screening approach, we adopted molecular docking and the MM/PB-SA method based on MD simulations as the first and second filters, respectively. First, we performed molecular docking by using the conformations of a target protein and the compounds contained in the compound library. Additionally, the results of molecular docking were applied to the post-processing for the selection of successfully docked compounds and the classification of multiple binding poses (see Materials and Methods). Next, all of the conformations obtained from the molecular docking were energy-minimized using molecular mechanics (MM) force-field (hereafter we call this MM calculations). MD simulations were then applied to multiple conformations of the protein-ligand complexes. The binding free energies were calculated by the MM/PB-SA method using the coordinate sets obtained from the MM calculations and MD simulations. Finally, we assessed the enrichment of active compounds by using ranked lists of compounds graded on the basis of their binding free energies.

## Results

To evaluate the ability of the MM/PB-SA method to act as a filter after molecular docking, we performed MD-based compound screening for four target proteins (trypsin, HIV-1 protease (HIV PR), acetylcholine esterase (AChE), and cyclin-dependent kinase 2 (CDK2)). These targets have been widely evaluated in structure-based computer-aided drug design [26], [29]–[34]. For each target protein, we first assessed the enrichment of 12 types of binding free energies (Table 1). These 12 types of binding free energies were classified into four categories. G01–G03 in category 1 were the energies calculated from the MM calculations. The other categories 2–4, which contained the energies calculated from the MD simulations, were classified according to the combination of coordinate sets used for the enthalpy calculations; G04–G06, G07–G09, and G10–G12 belonged to categories 2, 3, and 4, respectively (a detailed explanation is given in the Materials and Methods section.). Analyses of the Receiver Operating Characteristic (ROC) curves [35] are given in Table 2. An ROC curve is closely related to an enrichment curve but is not exactly equivalent to it. This curve describes the tradeoff between sensitivity and specificity. Sensitivity is defined as the ability of the classifier to detect true positives, while specificity is the ability to avoid false positives. The area under an ROC curve, i.e., the ROC value, indicates the quality of enrichment. The ROC value of a random classifier is 0.5, while that of an excellent classifier is greater than 0.9.

**Table 1:** Computational strategies of 12 binding free energies.

Category	Δ*G*_bind_	H_COMPLEX_	H_PROTEIN_	H_LIGAND_	TS_COMPLEX_	TS_PROTEIN_	TS_LIGAND_
1	G01	MM_COMPLEX_	MM_COMPLEX_	MM_COMPLEX_	–	–	–
	G02	MM_COMPLEX_	MM_COMPLEX_	MM_LIGAND_	–	–	–
	G03	MM_COMPLEX_	MM_PROTEIN_	MM_LIGAND_	–	–	–
2	G04	MD_COMPLEX_	MD_COMPLEX_	MD_COMPLEX_	TS_COMPLEX_	TS_COMPLEX_	TS_LIGAND_
	G05	MD_COMPLEX_	MD_COMPLEX_	MD_COMPLEX_	TS_COMPLEX_	TS_PROTEIN_	TS_LIGAND_
	G06	MD_COMPLEX_	MD_COMPLEX_	MD_COMPLEX_	–	–	–
3	G07	MD_COMPLEX_	MD_COMPLEX_	MD_LIGAND_	TS_COMPLEX_	TS_COMPLEX_	TS_LIGAND_
	G08	MD_COMPLEX_	MD_COMPLEX_	MD_LIGAND_	TS_COMPLEX_	TS_PROTEIN_	TS_LIGAND_
	G09	MD_COMPLEX_	MD_COMPLEX_	MD_LIGAND_	–	–	–
4	G10	MD_COMPLEX_	MD_PROTEIN_	MD_LIGAND_	TS_COMPLEX_	TS_COMPLEX_	TS_LIGAND_
	G11	MD_COMPLEX_	MD_PROTEIN_	MD_LIGAND_	TS_COMPLEX_	TS_PROTEIN_	TS_LIGAND_
	G12	MD_COMPLEX_	MD_PROTEIN_	MD_LIGAND_	–	–	–

We performed MM calculations (MM energy minimization) or MD simulations of a complex, a protein, and a ligand, and evaluated 12 types of binding free energies by combining the respective coordinate sets. The enthalpy contributions of *G*_protein_ and *G*_ligand_ in equation 2 were calculated in the following two ways: (1) by using the coordinate sets of a protein (or ligand) obtained from the MD simulations (or MM calculations) of the protein (or ligand) and (2) by using the coordinate sets extracted from the MD simulation of a complex. Similar to the enthalpy contribution, the entropy contribution was also calculated by combining the respective MD coordinate sets. H indicates the sum of <*E*_MM_>, <*G*_PB_>, and <*G*_SA_> in equation 3, and TS indicates the entropy term in equation 3. MD _COMPLEX_ (TS_COMPLEX_), MD_PROTEIN_ (TS_PROTEIN_), and MD_LIGAND_ (TS_LIGAND_) denote the use of MD coordinate sets for a complex, protein, and ligand, respectively. Similarly, MM_COMPLEX_, MM_PROTEIN_, and MM_LIGAND_ denote the use of MM coordinate sets for a complex, protein, and ligand, respectively.

**Table 2:** Area under ROC curves.

Δ*G*_bind_	Trypsin	HIV PR	AChE	CDK2	CDK2(l)
G01	0.754 (0.318)	0.775 (0.696)	0.655 (0.597)	0.719 (0.685)	0.719 (0.685)
G02	0.651 (0.323)	0.561 (0.550)	0.719 (0.627)	0.595 (0.652)	0.595 (0.652)
G03	0.491 (0.283)	0.538 (0.492)	0.747 (0.554)	0.586 (0.604)	0.586 (0.604)
G04	0.623 (0.321)	0.789 (0.435)	0.527 (0.409)	0.597 (0.599)	0.659 (0.627)
G05	0.539 (0.291)	0.775 (0.425)	0.506 (0.436)	0.565 (0.585)	0.636 (0.614)
G06	0.765 (0.391)	0.979 (0.550)	0.831 (0.603)	0.558 (0.568)	0.624 (0.558)
G07	0.543 (0.300)	0.514 (0.373)	0.509 (0.413)	0.625 (0.623)	0.647 (0.638)
G08	0.486 (0.265)	0.528 (0.383)	0.513 (0.435)	0.586 (0.606)	0.635 (0.626)
G09	0.694 (0.388)	0.778 (0.471)	0.843 (0.645)	0.606 (0.610)	0.622 (0.616)
G10	0.431 (0.369)	0.336 (0.373)	0.654 (0.458)	0.667 (0.615)	0.614 (0.606)
G11	0.422 (0.327)	0.326 (0.389)	0.678 (0.464)	0.653 (0.604)	0.605 (0.602)
G12	0.526 (0.406)	0.516 (0.352)	0.735 (0.541)	0.631 (0.605)	0.612 (0.606)

This table lists the ROC values obtained when the active compounds in the top 1,000 compounds are all considered to be as true positive. The values in parentheses denote the ROC values of a single pose, while the others denote those of multiple poses. The underlining indicates the highest ROC values in the respective categories. CDK2(l) indicates the values of longer MD simulations (1.4 ns) of CDK2.

Table 2 shows the ROC values for all of the target proteins. From these values, we can observe three common features for three of the target proteins (trypsin, HIV PR, and AChE), excluding CDK2. It is obvious that the ROC values for all of the binding free energies (G01–G12) of multiple poses are higher than those of a single pose, suggesting that docking and its post-processing can sample potentially correct docking poses of active compounds. This implies that the potentially correct binding mode is contained within the top 10 highest-scored docking poses but is not always the highest-scored docking pose. In our study, after docking and post-processing, MD simulations were applied to an average of 5–6 docking poses for each compound in order to increase the efficiency of the sampling of a ligand's conformations. Although MD simulations of multiple poses are expensive, they are necessary for improving enrichment.

**Figure 1:**
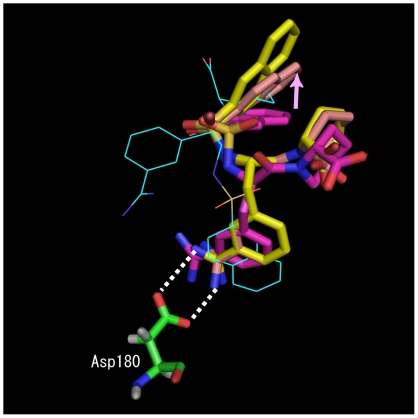
Successful example of MD simulations using multiple poses. The color codes for the stick models are as follows: yellow, conformation of the inhibitor in the crystal structure; purple, 7^th^-ranked docking pose; and pink, conformation of the 7^th^-ranked docking pose after the MD simulation. In addition, the highest-scored docking pose is shown by the wireframe model. The highest-scored docking pose (wireframe model) is inaccurate, but the 7^th^-ranked docking pose is similar to that of the crystal structure. MD simulation of the 7^th^-ranked pose improved the key hydrogen bonds and the position of the naphthalene group and G06 value of the 7^th^ ranked docking pose was the lowest in all the poses.

The second common feature is that the highest ROC value for each target protein was obtained for the energies calculated from the MD simulations, rather than for those calculated from the MM calculations. This implies that the introduction of protein flexibility and the effect of water molecules facilitated the refinement of the protein-ligand interactions and that the MD-based MM/PB-SA method provided a more reliable binding free energy. These two common features were clearly seen in the results for the active compounds. A typical successful example of MD simulations using multiple poses is shown in Figure 1. In the crystal structure of trypsin complexed with an inhibitor [36], the amidine group of the inhibitor (Figures 1 and S1; active compound (13) of trypsin) formed hydrogen bonds with the important residue Asp-180 in the binding pocket. Further, the highest-scored docking pose was so inaccurate that no important interactions were observed at all, but the 7^th^ ranked docking pose was similar to that of the crystal structure. In addition, the application of the MD simulation to the 7^th^ ranked docking pose appropriately improved the key hydrogen bonds and the position of the naphthalene group and G06 value of the 7^th^ ranked docking pose was the lowest in all the poses.

The last common feature was that the binding free energies with no entropy terms (i.e., G06, G09, and G12), which were obtained by using the trajectories of the MD simulations, showed the highest ROC values in the respective energy categories (2–4). Thus, the introduction of entropy terms tended to reduce enrichment. This is probably due to the difficulty of computing entropy values for the MM-PB/SA energy function. We will further discuss this problem in the Discussion section. Our MD simulations encouraged conformational relaxation, and the binding enthalpy from the MM-PB/SA method could satisfactorily increase the enrichment performance. However, the treatment of binding entropy terms involves certain unsolved problems. Here, we performed a statistical analysis using data on the ROC values to evaluate the differences between key classifiers, G01 (multiple poses), G06 (multiple poses), and molecular docking (Table 3). The program DBM MRMC version 2.1 was used in this analysis [37]–[41]. From this analysis, it was obvious that the differences in the ROC values between G06 and docking, and those between G06 and rescoring (docking), were statistically significant for trypsin and HIV PR, but the difference in the ROC values between G06 and docking for AChE was not statistically significant. On the other hand, the differences in the ROC values between G01 and docking were not statistically significant for trypsin, HIV PR, and AChE. An examination of the entire data set indicated that the binding free energies of multiple poses, especially G06, which was obtained from the MD trajectories of just the protein-ligand complexes with no entropies, showed a high and stable ability to enrich the active compounds.

**Table 3:** Statistical comparisons of ROC values.

	Trypsin	HIV PR	AChE	CDK2
G01#	0.754 (0.010)	0.775(0.054)	0.655(0.117)	0.719 (0.064)
G06#	0.765 (0.092)	0.979(0.004)	0.831(0.135)	0.558 (0.065)
GOLD/Rescore#	0.476(0.099)/0.477(0.081)	0.621(0.102)/0.636(0.117)	0.614(0.159)/0.268(0.098)	0.679 (0.067)/0.530 (0.038)
P values(G01/Docking)	0.054	0.542	0.922	0.915
p values(G01/Rescore)	0.055	0.320	0.019*	0.102
p values(G06/Docking)	0.045*	0.039*	0.330	-
p values(G06/Rescore)	0.046*	0.014*	0.001*	0.712

This analysis was analyzed by the program DBM MRMC 2.1 [37]–[41]. This program uses a jackknife method [37] to assess the statistical significance of the observed difference between two classifiers. The p-value (G06/Docking) in CDK2 is not shown because the ROC value of G06 was less than that of Docking. This analysis indicated that the differences between G01 and G06 were not statistically significant (data not shown). The analyses were performed using results of multiple poses. “Rescore” indicate the result of rescoring approach (docking) (see Materials and Methods).

# These values are ROC values. Standard errors are shown in parentheses.

*: Differences are considered statistically significant at p<0.05.

**Figure 2:**
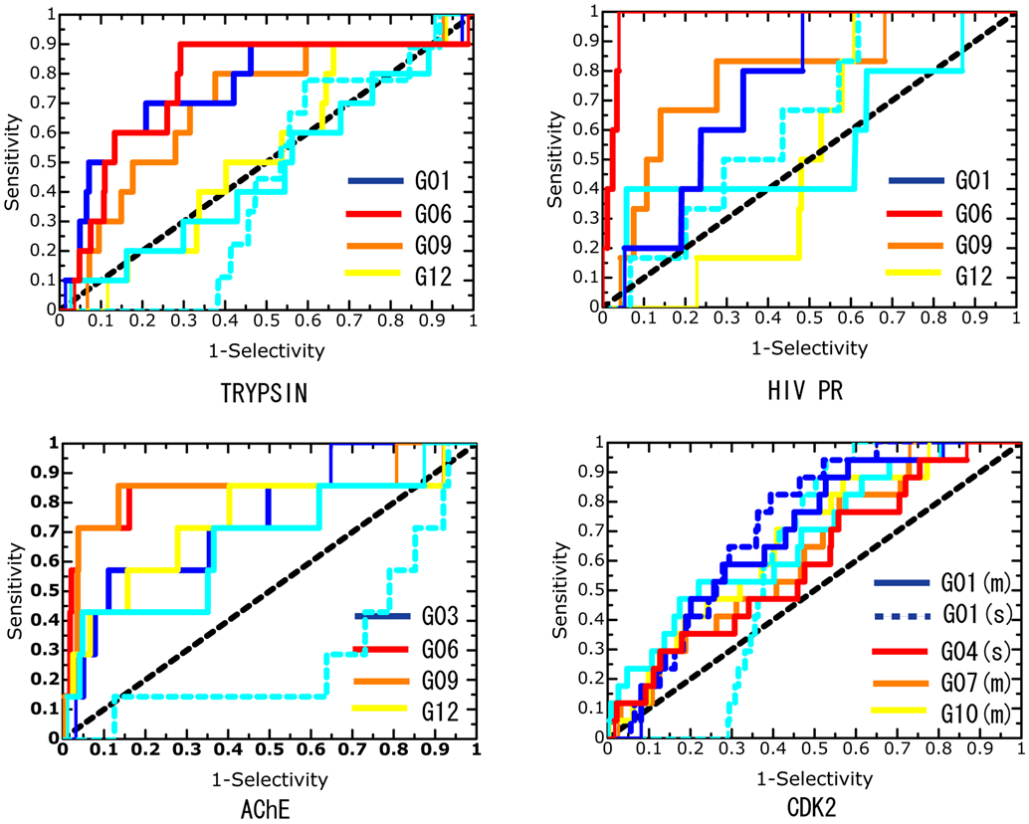
ROC curves for the four target proteins. Each graph shows the sensitivity versus 1-specificity. These indicate the ROC curves obtained when the active compounds in the top 1,000 compounds are all considered to be as true positive. The black dashed, sky blue solid, and sky blue dashed lines indicate random screening, molecular docking and rescoring (docking), respectively. For trypsin, HIV PR, and AChE, the ROC curves of the binding free energies (multiple poses) with the highest ROC values in the respective categories are shown. For CDK2, the curves of G01 (single and multiple poses), G04 (single pose), G07 (multiple poses), and G10 (multiple poses) are shown. (s) and (m) indicate single pose and multiple poses.

This paper provides a detailed account of the ability of our approach to discriminate active compounds from inactive ones. Figure 2 shows the ROC curves for the respective target proteins. The ROC curves of the binding free energies, with the highest ROC values in the respective categories, were observed for trypsin, HIV PR, and AChE. Table 4 shows the information on the enrichment factors to allow the abilities of the classifiers to be understood clearly.

**Table 4:** Enrichment factors for top 1,000 compounds.

	Trypsin	HIV PR	AChE	CDK2
EF (10%)	5.00 (G01)	3.33 (G01)	4.29 (G03)	1.76 (G01)
	4.00 (G06)	10.0 (G06)	7.14 (G06)	1.76 (G04)
	3.00 (G09)	3.33 (G09)	7.14 (G09)	1.43 (G07)
	0.00 (G12)	0.00 (G12)	4.29 (G12)	2.14 (G10)
	1.00 (Docking)	5.00 (Docking)	4.29 (Docking)	2.35 (Docking)
	0.00 (Rescoring)	1.67(Rescoring)	0.00 (Rescoring)	0.00 (Rescoring)
EF (30%)	2.33 (G01)	2.22 (G01)	1.90 (G03)	1.96 (G01)
	3.00 (G06)	3.33 (G06)	2.85 (G06)	1.17 (G04)
	2.00 (G09)	2.78 (G09)	2.85 (G09)	1.67 (G07)
	0.66 (G12)	0.55 (G12)	2.38 (G12)	1.90 (G10)
	1.00 (Docking)	2.78 (Docking)	1.43 (Docking)	1.76 (Docking)
	0.33 (Rescoring)	1.67 (Rescoring)	0.48 (Rescoring)	0.19 (Rescoring)

The enrichment factor (EF) can be defined as:
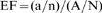
where a is the number of active compounds in the n top-ranked compounds and A is the number of total N compounds. In this table, the n for EF (10%), n for EF (30%), and N were 100, 300, and 1,000, respectively. For the respective proteins, A, n for EF (10%), and n for EF (30%) indicate the numbers of active compounds in the top 1,000, top 100, and top 300, respectively. All of the EF values calculated from the result of screening using multiple poses. “Rescoring” indicate the result of rescoring approach (docking) (see Materials and Methods).

For trypsin, 10 active compounds (out of 21) were ranked in the top 1,000 compounds (Figure S1). No significant difference was observed in the results of molecular docking and random screening for these top 1,000 compounds. Considerable improvement was observed in the results of the MM calculations and MD simulations. G01 and G06, in particular, showed high enrichment performances, and the enrichment factors for the top 100 compounds were 5.00 and 4.00, respectively (see Table 4). Furthermore, G06 detected no less than nine active compounds in the top 300.

For HIV PR, 6 active compounds (out of 8) were ranked in the top 1,000 compounds (Figure S2). A slightly better enrichment was achieved by docking than by random screening. As seen in the curves, we found drastically improved enrichment by G06. It detected 6 active compounds in the top 100 compounds, and the enrichment factor for the top 100 was 10.0 (Table 4).

For AChE, 7 active compounds (out of 14) were detected in the top 1,000 compounds (Figure S3). We found that the enrichments of G06 and G09 were considerably better than that of molecular docking, although the difference in the ROC values between G06 (or G09) and docking was not statistically significant. Because there was only a slight difference between G06 and G09, both of them detected five active compounds in the top 100.

For CDK2, the ROC curves of the following representative binding free energies were drawn: G01 (single and multiple poses), G04 (single pose), G07 (multiple poses), and G10 (multiple poses). Seventeen active compounds (out of 26) were ranked in the top-scoring 1,000 compounds (Figure S4). The G01 of single and multiple poses as obtained from the MM calculations showed higher enrichment than random screening; however, there was no statistically significant difference between the results of G01 and molecular docking (see Table 3). The G01 of single and multiple poses detected 10 active compounds in the top 300 and showed only slightly higher enrichment factors than molecular docking (Table 4). In contrast, G04, G07, and G10, which were obtained from the MD simulations, remained unchanged or worsened as compared to docking, although they identified 6 or 7 active compounds in the top 200. Over all, the enrichments for CDK2 were not at all improved as mentioned above.

The ROC values for CDK2 showed a different tendency as compared to those for the other three proteins (Table 2). Among the 12 types of energies, the G01 of single and multiple poses showed the highest values (0.685 and 0.719, respectively), which implies that the enrichments of the MM calculations were better than those of the MD simulations. Moreover, for 7 types of energies (out of 12), the single pose results showed higher enrichment than those of multiple poses. In addition, the binding free energies with entropy terms showed slightly high enrichment performances in the respective categories (2–4), which were calculated from MD simulations. In particular, G04, G07, and G10, which included the binding entropy effects of the ligands, showed the highest ROC values in their respective categories.

**Figure 3:**
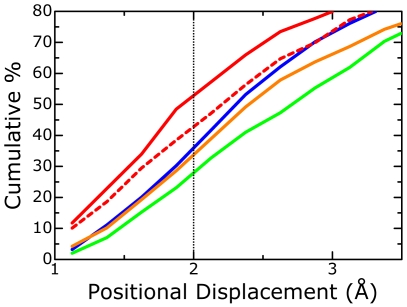
Mobility of ligand molecules in MD simulations. This graph indicates cumulative percentage graph of positional displacements of ligand molecules relative to the respective proteins between docking structures and final MD structures. The blue, green, orange, and red solid lines indicate cumulative curves for trypsin, HIV PR, AChE, and CDK2, respectively. The broken line indicates a curve for longer MD simulations (1.4 ns) of CDK2. From the red solid line, it is clear that the docked ligands for CDK2 did not move in the MD simulations since the positional displacements of approximately 50% of the ligand conformations were less than 2.0 Å.

We monitored the mobility of ligand molecules in MD simulations of CDK2. Figure 3 shows the cumulative percentages of positional displacements of ligand molecules relative to each protein between the docking and final MD structures. From this figure, it is clear that the docked ligands for CDK2 did not move very much in the MD simulations, as compared to the other target proteins, which implies that the protein-ligand interactions were not fully relaxed. Such insufficiency in conformational relaxation/refinement directly influences protein-ligand interactions. Particularly for active compounds, the binding modes obtained by MD simulations were some different from those of experimental structures (refer to Figures S5 and S6). These data suggested that the use of MD simulations for CDK2 led to structural uncertainties for active compounds. In addition, the interactions of the inactive (decoy) compounds would not be refined fully in MD simulations. We think that such low mobility for ligand molecules and the improper conformational dynamics are due to an improper MD setup. This would be the reason why the enrichments of the MD simulations using multiple poses were worse than those of the MM calculations for CDK2.

## Discussion

We evaluated the ability to enrich active compounds for four target proteins: trypsin, HIV PR, AChE, and CDK2. Our screening approach could improve the molecular docking results for all of the proteins except CDK2. For trypsin, HIV PR, and AChE, our results indicated that the use of multiple poses improved the enrichments of all the MM calculations and MD simulations. In addition, the binding free energies calculated from the MD simulations showed higher and more stable enrichments than those of the docking and MM calculations. In particular, the G06 using multiple poses was considered to be effective. This energy contained no entropy components. Further, the enthalpy components were calculated using the coordinate sets extracted from the MD simulation of a complex.

Kuhn and coworkers [25] reported that for the MM/PB-SA values of MM calculations, the strategy of using multiple poses could only show a high enrichment when the correct binding mode was contained within the higher-scored docking conformations, but was not captured with a single pose. In our study, we carefully selected multiple docking poses by the post-processing of docking results and used an average of five to six docking poses for each compound. As a result, the correct binding modes or potentially correct modes that could be refined by the MD simulations were sampled within the selected multiple docking poses, which did not often correspond to the top-scored pose. Therefore, the results using multiple poses showed a higher enrichment than those obtained using a single pose.

In addition, Kuhn et al. [25] showed that the use of MD simulations often leads to structural uncertainties and an inaccurate estimation of the binding free energy. The MM/PB-SA energies of the MM calculations and MD simulations in their study corresponded to G01 and G04 in our study. A comparison between G01 and G04 indicated that the enrichment of G04 was lower than that of G01, which is consistent with the results of Kuhn and coworkers [25], although there were large differences in the MD setup, MM/PB-SA setup, and target proteins. G01 contained only the enthalpy components that were calculated using the coordinate sets derived from the MM calculation of a complex. G04 contained the binding entropy effect of the ligand. Further, the enthalpy components were calculated using the coordinate sets from the MD calculation of a complex. The difference between G04 and G06 was the presence of the entropy effect. Therefore, we consider that for trypsin, HIV PR, and AChE, the structural refinement/relaxation by longer and higher time resolution MD simulations and the relatively accurate estimation of binding free energy (enthalpy) by the MM/PB-SA method led to increased enrichment, but the introduction of the entropy values induced an uncertainty in the binding free energies. On the other hand, we think that the use of MD simulations for CDK2 led to structural uncertainties and then an inaccurate estimation of the binding free energy (Figures S5 and S6). This would be due to an improper MD setup, as Kuhn and co-workers suggested in their paper [25].

Basically, it is well-known that it is difficult to calculate entropy values properly. In our work, the entropy values were calculated by principal component analysis (PCA). These values are sensitive to the data sampling frequency [42],[43] and are likely to be overestimated [44]. Therefore, we believe that the entropy values were slightly unstable and not completely reliable. An alternative computational method is normal mode analysis. This may be stable to some extent, but it is known that conformations at different local energy minima provide rather similar entropy values even though there are differences in the finite temperature [42]. Moreover, the computational cost is significantly high to use for the calculation of many structures. Thus, even if we were to use normal mode analysis, the entropy values would induce an uncertainty in the binding free energies. Therefore, in order to achieve further improved enrichment, it is necessary to improve the calculations for the entropy terms.

**Figure 4:**
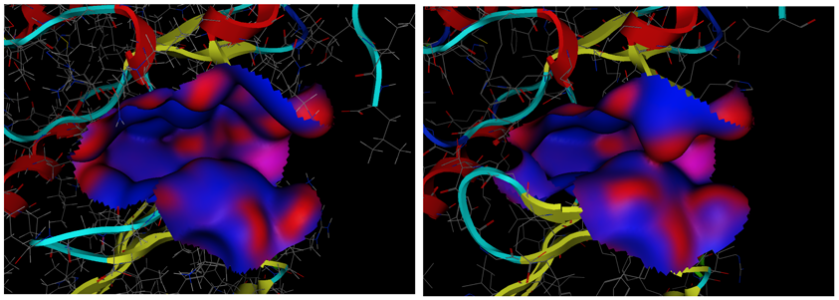
Binding pockets in two different X-ray crystal structures for CDK2. The left figure indicates the binding pocket in the CDK2-Oxindole inhibitor complex (PDB Id: 1FVV). This structure was used in our study. The right figure indicates the binding pocket in the CDK2-NU2058 inhibitor complex (PDB Id: 1H1P). The hydrophobic regions in the binding pocket are drawn in blue, and the hydrophilic regions are drawn in red. From this figure, it is clear that large hydrophobic regions were located in both binding pockets. In contrast, the shapes of the two binding pockets were considerably different, which means that the binding pocket of CDK2 was flexible.

**Figure 5:**
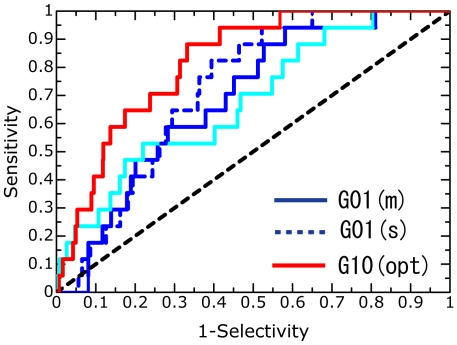
ROC curves of the LR-MM/PB-SA approach for CDK2. This graph shows the sensitivity versus 1-specificity. This indicates the ROC curves obtained when the active compounds in the top 1,000 compounds are all considered to be as true positive. The black dashed, sky blue solid, and sky blue dashed lines indicate random screening, molecular docking and rescoring (docking), respectively. The G01(m), G01(s), molecular docking and rescoring(docking) are the same as in Figure 2. G10 (opt) is the ROC curve of G10 (multiple poses) obtained by the LR-MM/PB-SA approach. The ROC value is 0.812.

Our strategy could not significantly improve the molecular docking results for CDK2. It is well known that, as compared to the binding pockets of the other three proteins, the binding pocket of CDK2 is more flexible and hydrophobic. We compared the binding pockets in two different X-ray crystal structures of CDK2 [45],[46] (Figure 4). This figure indicates that the shape of the binding pocket is very flexible and that the hydrophobic region covers the surface of the binding pocket. In addition, a study on molecular docking using different CDK2 crystal structures reported that the volume (flexibility) of the binding site is a key factor for predicting docking poses [29]. Although only one CDK2 structure was used in this study, we applied MD simulations to protein-ligand structures obtained from molecular docking to facilitate the relaxation of protein-ligand interactions. Unfortunately, our MD simulations were insufficient to relax the protein-ligand conformations in the binding pockets (see Figure 3). Such insufficiency is believed to be due to the MD setup. To improve the insufficient relaxation, we applied the MD simulations for 1.4 ns to each configuration; this simulation time was twice that of the initial MD simulation time. These MD simulations effected some relaxation/refinement of the ligand conformations (Figure 3); the enrichments of the multiple poses were found to be higher than those of the single pose (Table 2). Despite this, G04–G09 showed only small improvements in the enrichment performance. These results suggest that further improvement of the MD setup was necessary. To obtain information about how to improve the MD setup, we attempted to maximize the ROC values by using an approach based on the linear response (LR) [47] and MM/PB-SA methods (LR-MM/PB-SA approach [48]) The LR-MM/PB-SA equation was derived from equations 2–4 (see Materials and Methods):
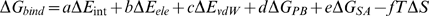
(1)where a, b, c, d, e, and f are weighting factors ranging from 0.5 to 1.5. The terms on the right side of equation 1 represent the energy difference between the complex and protein plus ligand. This approach is usually used for estimating the binding affinity by combining an empirical MM/PB-SA energy calculation with an LR optimization of coefficients against the experimental binding affinities of several compounds. The optimized free energy model is used for interpreting the binding model and predicting the binding affinity of unknown molecules. In our study, we optimized the weighting factors to maximize the ROC value, that is, the enrichment performance, using a genetic algorithm (GA). We applied the LR-MM/PB-SA approach to the G10 of multiple poses obtained from the initial MD simulations, because G10 showed the highest ROC value among those of the binding free energies calculated from the MD simulations (Table 2). As a result, when the weighting factors of a–f were 1.12, 0.91, 1.47, 1.01, 0.87, and 1.49, respectively, a maximum ROC value of 0.812 was obtained (Figure 5). This result suggested that the LR-MM/PB-SA approach was effective at improving the enrichment performance, and these weighting factors indicated an improvement plan for the MD setup. The weighting factor of the entropy term, 1.49, would contribute to dilute the percentage of inactive (decoy) compounds. This would be related to the fact that the ligands in the binding pocket of CDK2 could not move largely (Figure 3). In addition, it is conceivable that the weighting factor of Δ*E*_vdW_, i.e., 1.47, enriched the active compounds because they include a hydrophobic region and formed comparatively strong hydrophobic interactions with the binding pocket (See Figure S4). As the binding modes obtained through MD simulations were some different from the experimentally observed binding modes, the conformational refinement was considered insufficient or improper to accurately predict the binding free energy. This information also suggests that fully conformational relaxation/refinement is required to improve the enrichment performance. In this study, the ligand, water molecules, and protein residues around the binding pocket were allowed to move, but other protein residues were restrained to the X-ray structure in all of the MM calculations and MD simulations (a detailed explanation is given in the Materials and Methods section). Hence, to achieve the conformational relaxation of the binding pocket of CDK2, allowing wider protein residues to move in MD simulations and longer MD simulations are required. The former is an especially important parameter for improving the enrichment performance, although it would increase the computational cost. In our next study, which will focus on the extent of the mobility of protein residues, along with simulation time and force-field parameters for organic small molecules, we will attempt to optimize the MD setup using a recent widely used dataset of decoy compounds [49].

The computational screening of large compound libraries involves the use of hierarchical multiple filters, such as ligand- and structure-based approaches. Molecular docking plays the primary role in these filters. With advancements in computer performance and computational chemistry, docking programs have become more accurate, but their ability to enrich hit compounds remains unsatisfactory. In order to improve the enrichment performance of molecular docking, we attempted to use the MM/PB-SA method [50] as a post-molecular docking filter. The basis of our approach was to perform massive MD simulations of protein-ligand conformations obtained from molecular docking, aim at the refinement/relaxation of protein-ligand conformations after docking, and predict more accurate binding free energies using the MM/PB-SA method in a practical time for lead discovery. Combining molecular docking and MD simulations basically allows each of them to neutralize the other's defects, but certain problems remain even with MD simulations, particularly with regard to compound screening applications. The major drawback of MD simulations is insufficient sampling due to the significant computational cost involved. To solve this problem, we performed MD simulations using various docking conformations obtained by molecular docking. However, the computational cost of this technique was approximately five to six times that of MD simulations using single docking conformations, such as the top-scored docking conformation. The enormous computational time needed for MD simulations is a serious problem. Here, we solved this problem by accelerating most of the time-consuming operations of the MD simulation using a high-performance special-purpose computer for MD simulations, “MDGRAPE-3” [27],[28]. Accordingly, our approach could be performed in a practical time (about a week) for lead discovery. The evaluation in this study provides valuable information on in-silico drug design. Further, a more rigorous MD-based filter is under consideration for further improving the enrichment performance. This technique will also be applied to the lead optimization stage of drug development research.

In conclusion, our approach could improve the enrichment of virtual screening by molecular docking. Among the 12 types of binding free energies, G06, which was obtained from the MD simulations using multiple poses, showed the highest and most stable ability to enrich the active compounds. The strategy of multiple poses can be used to sample the potentially correct poses of active compounds; thus, it increases the enrichment performance. Since the G06 enrichment factors for the top 100 compounds ranged from 4 to 10 (see Table 4), which indicates approximately 1.6–4.0 times higher values than the enrichment performance of molecular docking, with the exception of CDK2, it is obvious that a stable and high enrichment can be achieved after molecular docking. In addition, G06 is suitable for compound screening because its computational cost is the least among those of the other MM/PB-SA energies obtained from the MD simulations. We also confirmed that G01, which was obtained from the MM calculations, showed good enrichment ability despite its low computational cost. This result agreed with that of the previous study [25]. The ability of G01 to enrich active compounds was lower and less stable than that of G06, but we believe that G01 acted as an effective filter between molecular docking and the MD-based MM/PB-SA method. From this study, we conclude that the application of MD simulations to virtual screening for lead discovery is effective and practical, but that further optimization of the MD simulation protocols is required for the screening of various target proteins, including kinases.

## Materials and Methods

### Preparation of the Target Protein

We applied our approach to four target proteins: trypsin, HIV PR, AChE, and CDK2. These structures with crystallographic resolutions of less than 3.0 Å, were retrieved from the Protein Data Bank (PDB) because the conformations of residues in the binding pocket affect the molecular docking results (PDB Id: 1C5S (trypsin) [51], 1HWR (HIV PR) [52], 1E66 (AChE) [53], and 1FVV (CDK2) [46]). All of the bound crystal water molecules, ligands, and other organic compounds were removed from each protein. Hydrogen atoms were added, and energy minimizations on the hydrogen atoms were performed using the Molecular Operating Environment (MOE) program (Chemical Computing Group Inc. [54]).

### Seeded Compound Library for Docking

For each target protein, we prepared a test set of compounds that included 10,000 randomly selected compounds, or decoys, from the Maybridge library of compounds and experimentally known active compounds. It was confirmed that 95.5% of the selected decoy compounds obeyed the Lipinski rule of 5 [55]. The active compounds, which had binding affinities (*K*_i_, *K*_d_, or IC_50_) below 30 µm, were selected from the PDBbind database [56],[57] and by referring to the literatures [26],[58]. Most of the active compounds also obeyed the Lipinski rule of 5. The numbers of active compounds selected for each of the respective target proteins was as follows: 21 (trypsin), 8 (HIV PR), 14 (AChE), and 26 (CDK2) (see Figure S1, S2, S3, S4). For each compound of the test set, a 3D conformation was generated, ionized, and energy minimized using LigPrep (Schrödinger Inc. [59]), assuming a pH of 7.0.

### Docking

Molecular dockings were performed using the Genetic Optimisation of Ligand Docking (GOLD) version 3.1 [9],[10]. This program employs a GA to explore the possible binding modes. The standard default settings for the GA parameters were used. The binding site radius was 12 Å. We performed the docking run three or four times using the GoldScore or ChemScore function for each target protein and selected the result that showed the best enrichment. GoldScore (default settings) was used as the scoring function for trypsin and HIV PR. In contrast, ChemScore (default settings) was used for AChE and CDK2 because docking runs using GoldScore can detect few of the successfully docked active compounds for AChE and CDK2. For AChE alone, the torsional rotations of Phe-330 (chi1 and chi2) were treated as flexible in the docking process. For each docking run, the 10 highest-scoring docking poses were saved to obtain a variety of binding modes.

### Post-processing of the Docking Results

First, among the 10 highest-scoring docking poses saved for each compound, those in which the compound did not occupy the binding pocket or did not interact with the important residues were removed. The latter was used only for trypsin and HIV PR. The important residues were Asp180 for trypsin and Asp24 in each monomer for HIV PR. These treatments had the effect of reducing the false positives for molecular docking. The docked compounds were then arranged in descending order from the highest score with respect to the multiple docking poses, and the top 1,000 compounds were selected from the test set. Finally, for the top 1,000 compounds, the docking poses of each compound were clustered using the root mean square deviation of 0.9 Å (complete link method [60]). After post-processing, approximately 6,000 docking poses were selected for the 1,000 compounds, which were then used as the initial conformations for MD simulations. Some active compounds were not ranked in the top 1,000. The numbers of active compounds in the top-scoring 1,000 were 10, 6, 7, and 17 for trypsin, HIV PR, AChE, and CDK2, respectively. In addition, the compounds in the top-scoring 1,000 were rescored with ChemScore (trypsin and HIV PR) or GoldScore (AChE and CDK2) because it is known that the rescoring approach increases the enrichment performance [61]. Furthermore, we analyzed ROC curves using molecular weight as classifier (Figure S7). From statistical analysis, it is obvious that the differences in the ROC values between G06 and molecular weight were statistically significant for trypsin, HIV PR, AChE.

### MD Simulation Protocols

**Figure 6:**
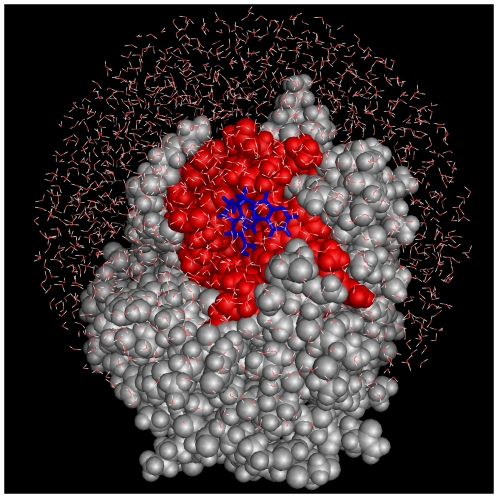
System for MD simulation of trypsin. The protein is shown by the space-filled model, and the ligand is colored blue. The peripheral residues around the active center (red region), a ligand, and water molecules were allowed to move in the MD simulation. The protein residues (grey) were restrained to the X-ray structure by a harmonic energy term. Similar systems were used for the other target proteins.

We performed MD simulations of each complex (ligand-bound protein), protein, and ligand to obtain various types of binding free energies (see the following subsection). The active sites of the protein-ligand complexes were immersed in an approximately 28–30 Å sphere of transferable intermolecular potential 3 point (TIP3P) water [62] molecules. The radius of the water droplet was selected such that the distance of the atoms of all the docked compounds from the water wall was greater than 15 Å (see Figure 6). The total number of atoms in the respective systems was approximately 8,000–12,000. On the solvent boundary, a half-harmonic potential (1.5 kcal/mol-Å^2^) was applied to prevent the evaporation of the water molecules. The ligand, water molecules, and protein residues that were approximately 12 Å of the active center were allowed to move, but other protein residues were restrained to the X-ray structure by the harmonic energy term (1.5 kcal/mol-Å^2^) in all of the MM calculations, namely the MM energy-minimization, and MD simulations. For the simulations of the ligands, each ligand was immersed in a water droplet, and this structure was used as the initial structure for the MD simulation of the ligand. In addition, the simulation of each protein (trypsin, HIV PR, AChE, and CDK2) was performed in the same manner as that of the complex.

All of the simulations were performed using AMBER 8.0 [63] modified for MDGRAPE-3 [27],[28]. The ff03 force field [64] was adopted, and the time step was set at 0.5 fs. To carefully consider the motion of hydrogen atoms in the interactions between the ligands and protein residues, no bond length constraint was applied to solute atoms. The temperature of each system was gradually increased to 300 K during the first 25 ps, and additional MD simulations were performed for 700 ps for equilibration. The temperature was maintained at 300 K by using the method described by Berendsen et al. [65], and the system was coupled to a temperature bath with coupling constants of 0.2 ps. The parameters and charges for the ligands were determined using the *antechamber* module version 1.27 of AMBER 8.0 [63] by utilizing the general atom force field (GAFF) [66] and the AM1-BCC charge method [67],[68]. Although the computational cost of the AM1-BCC charge method is low, a some difference between the charge and that of ff03 was noticeable. Since the original GAFF parameters were insufficient to cope with the parameters of all the ligands, we filled the missing parameters on the basis of the information on regarding atom types, bonds, valences, angles, and dihedrals by using an in-house program (see Text S1). (Note: these parameters for proteins and small organic molecules are very important to calculate the binding free energies between proteins and ligands)

Our MDGRAPE-3 system is a cluster of personal computers, each equipped with two MDGRAPE-3 boards. Each board contains 12 MDGRAPE-3 chips and has a peak speed of approximately 2 Tflops. The computations of non-bonded forces and energies for MD simulations were accelerated by MDGRAPE-3, and the other calculations were performed by the host central processing unit (CPU). In this study, we used 50 host computers equipped with 100 MDGRAPE-3 boards. The calculations for an MD simulation and the estimation of the binding free energies by the MM/PB-SA method were performed simultaneously. The average computational time for a single protein-ligand complex was 2.5 h, and the computations for approximately 6,000 protein-ligand conformations obtained by docking for each protein were completed in a week. The total simulation time for each protein was 4 µs, which corresponded to an 8-µs MD simulation with a standard time step of 1 fs. A single MD simulation for the system (Figure 6), without using MDGRAPE-3, requires more than 10 times the abovementioned computational time. Thus, in the current state, it would be quite difficult to use our screening approach without the MDGRAPE-3 system in a practically appropriate time for lead discovery. Therefore, our study can provide important information for MD-based screening.

### Calculation of Binding Free Energy by the MM/PB-SA Method

The production MD trajectory was collected for the last period of 210 ps. In the calculation of the binding free energies by the MM-PB/SA method, the water molecules were replaced with implicit solvation models. The binding free energy was calculated by the following equations.
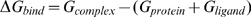
(2)

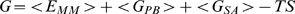
(3)

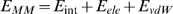
(4)

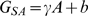
(5)In the above equations, < > denotes the average for a set of 30 conformations along an MD trajectory. *E*_int_ includes the bond, angle, and torsional angle energies; *E*_ele_ and *E*_vdW_ represent the intermolecular electrostatic and van der Waals energies, respectively. *G*_PB_ was calculated by solving the PB equation with the DelPhi program [69],[70], using the PARSE radii [71],[72] and AMBER charges. The grid spacing used was 0.5 Å. The dielectric constants inside and outside the molecule were 1.0 and 80.0, respectively. In equation 5, which calculates the nonpolar solvation contribution, *A* is the solvent-accessible surface area that was calculated using the Michael Sanner's Molecular Surface (MSMS) program [73], and *γ* and *b* are 0.00542 kcal/mol-Å^2^ and 0.92 kcal/mol, respectively. The probe radius was 1.4 Å. The conformational entropy term of the solute, *TS*, was approximated by a combination of a classical statistics expression and PCA [74], using the PTRAJ module of AMBER 8.0 [63]. In the PCA calculation, the last 210 ps (3,000 conformations) of each production trajectory were used.

The analysis of the binding free energy involved the calculation of the energies for conformations obtained from the MM (namely, energy-minimized) coordinates or MD trajectories. When the MM calculations or MD simulations of a complex, protein, and ligand were performed, we could obtain various types of binding free energies by combining the respective coordinate sets. The enthalpy contributions of *G*_protein_ and *G*_ligand_ in equation 2 were calculated in the following 2 ways: (1) by using the coordinate sets of a protein (or ligand) obtained from the MD simulations (or MM calculations) of the protein (or ligand) and (2) by using the coordinate sets extracted from the MD simulation of a complex. Similar to the enthalpy contribution, the entropy contribution was calculated by using the MD trajectories. When the entropy contributions of *G*_complex_, *G*_protein_, and *G*_ligand_ were calculated by using the MD trajectory of only the complex, we considered the entropy contribution of Δ*G*_bind_ to be zero because the energy components were almost cancelled. In this study, in order to thoroughly investigate which MM/PB-SA energies were suitable for compound screening, we adopted 12 binding free energies, G01–G12, to manage the entropy contributions independently of the enthalpy contributions (see Table 1). It should be noted that the coordinate sets for calculating the entropy contributions were not always consistent with those for calculating enthalpy contributions. Table 1 shows the enthalpy and entropy terms for computing of *G*_complex_, *G*_protein_, and *G*_ligand_ in equation 2. We classified the 12 binding free energies into four categories. Category 1 contained the energies obtained by the MM calculations, and categories 2, 3, and 4 contained those obtained by MD calculations. These categories were classified according to the combination of coordinate sets used for enthalpy calculations: G01–G03, G04–G06, G07–G09, and G10–G12 belonged to categories 1, 2, 3, and 4, respectively. Each binding free energy of a ligand adopts the minimum energies from among the energies of multiple poses. Thus, by gathering and arranging their energies, we were able to assess the enrichment performance of the screening approach.

## Supporting Information

Figure S1 Active compounds of trypsin. The structural formulae and PDB ids of active compounds used in the seeded compound library are shown in the following figures. The asterisks represent the active compounds in top-scoring 1,000.
(0.08 MB DOC)

Figure S2 Active compounds of HIV PR. The structural formulae and PDB ids of active compounds used in the seeded compound library are shown in the following figures. The asterisks represent the active compounds in top-scoring 1,000.
(0.53 MB DOC)

Figure S3 Active compounds of AChE. The structural formulae and PDB ids of active compounds used in the seeded compound library are shown in the following figures. The asterisks represent the active compounds in top-scoring 1,000.
(0.06 MB DOC)

Figure S4 Active compounds of CDK2. The structural formulae and PDB ids of active compounds used in the seeded compound library are shown in the following figures. The asterisks represent the active compounds in top-scoring 1,000. Compounds 1 and 19 were selected by referencing literatures.
(0.11 MB DOC)

Figure S5 Number of correctly docked conformations in top-scored active compounds. These indicate the number of correctly docked conformations in the top-scoring poses for active compounds obtained from molecular docking, G01, and G06. The red and blue bars indicate the number of poses within the root mean square deviations (RMSDs) of 2.5 and 3.5 Å from those of the experimental structure, respectively. The active compounds in the top 1,000 were investigated. In G06, the final MD structure was used.
(0.10 MB DOC)

Figure S6 Minimal RMSD values of computed poses from experimental poses for active compounds. The horizontal axis indicates the index number of active compounds in the top 1,000 shown in Figures S1, S2, S3, S4 and the vertical axis indicates the minimal RMSD among all the poses. For each protein, the poses obtained from molecular docking, G01, and G06 were investigated. In G06, the final MD structure was used. The red bars indicate the pose within the top-three scoring. For trypsin, HIV PR, and AChE, it was found that MD simulations could improve the binding modes and predict better binding free energies. For CDK2, however, it is suggested that MD simulations lead to structural uncertainties and an inaccurate estimation of the binding free energy.
(0.24 MB DOC)

Figure S7 ROC curves using molecular weight as classifier. This graph shows the sensitivity versus 1-specificity. This indicates ROC curves when the active compounds in the top 1,000 compounds are considered as total the true positives. ROC curves for trypsin, HIV PR, AChE, and CDK2 were drawn in blue, red, yellow, and orange, respectively. These ROC values for trypsin, HIV PR, AChE, and CDK2 are 0.454, 0.674, 0.462, and 0.430. From statistical analysis, it is obvious that the differences in the ROC values between G06 and molecular weight were statistically significant for trypsin, HIV PR, AChE. The differences in the ROC values between molecular docking and molecular weight were not statistically significant for all proteins.
(0.08 MB DOC)

Text S1 Assignment of missing force field parameters. We filled in the following missing parameters on the basis of the information on regarding atom types, bonds, valences, angles, and dihedrals by using an in-house program.
(0.06 MB DOC)

## References

[1] Young K, Lin S, Sun L, Lee E, Modi M (1998). Identification of a calcium channel modulator using a high throughput yeast two-hybrid screen.. Nat Biotechnol.

[2] Hamasaki K, Rando RR (1998). A high-throughput fluorescence screen to monitor the specific binding of antagonists to RNA targets.. Anal Biochem.

[3] Moore KJ, Turconi S, Miles-Williams A, Djaballah H, Hurskainen P (1999). A Homogenous 384-Well High Throughput Screen for Novel Tumor Necrosis Factor Receptor: Ligand Interactions Using Time Resolved Energy Transfer.. J Biomol Screen.

[4] Dunn D, Orlowski M, McCoy P, Gastgeb F, Appell K (2000). Ultra-high throughput screen of two-million-member combinatorial compound collection in a miniaturized, 1536-well assay format.. J Biomol Screen.

[5] Doman TN, McGovern SL, Witherbee BJ, Kasten TP, Kurumbail R (2002). Molecular docking and high-throughput screening for novel inhibitors of protein tyrosine phosphatase-1B.. J Med Chem.

[6] Chen J, Zhang Z, Stebbins JL, Zhang X, Hoffman R (2007). A fragment-based approach for the discovery of isoform-specific p38alpha inhibitors.. ACS Chem Biol.

[7] Carr RA, Congreve M, Murray CW, Rees DC (2005). Fragment-based lead discovery: leads by design.. Drug Discov Today.

[8] Hann MM, Leach AR, Harper G (2001). Molecular complexity and its impact on the probability of finding leads for drug discovery.. J Chem Inf Comput Sci.

[9] Jones G, Willett P, Glen RC (1995). Molecular recognition of receptor sites using a genetic algorithm with a description of desolvation.. J Mol Biol.

[10] Jones G, Willett P, Glen RC, Leach AR, Taylor R (1997). Development and validation of a genetic algorithm for flexible docking.. J Mol Biol.

[11] Ewing TJ, Makino S, Skillman AG, Kuntz ID (2001). DOCK 4.0: search strategies for automated molecular docking of flexible molecule databases.. J Comput Aided Mol Des.

[12] Goodsell DS, Morris GM, Olson AJ (1996). Automated docking of flexible ligands: applications of AutoDock.. J Mol Recognit.

[13] Halgren TA, Murphy RB, Friesner RA, Beard HS, Frye LL (2004). Glide: a new approach for rapid, accurate docking and scoring. 2. Enrichment factors in database screening.. J Med Chem.

[14] Friesner RA, Banks JL, Murphy RB, Halgren TA, Klicic JJ (2004). Glide: a new approach for rapid, accurate docking and scoring. 1. Method and assessment of docking accuracy.. J Med Chem.

[15] Rarey M, Kramer B, Lengauer T, Klebe G (1996). A fast flexible docking method using an incremental construction algorithm.. J Mol Biol.

[16] Bursulaya BD, Totrov M, Abagyan R, Brooks CL (2003). Comparative study of several algorithms for flexible ligand docking.. J Comput Aided Mol Des.

[17] Stahl M, Rarey M (2001). Detailed analysis of scoring functions for virtual screening.. J Med Chem.

[18] Wyss PC, Gerber P, Hartman PG, Hubschwerlen C, Locher H (2003). Novel dihydrofolate reductase inhibitors. Structure-based versus diversity-based library design and high-throughput synthesis and screening.. J Med Chem.

[19] Pearlman DA, Charifson PS (2001). Are free energy calculations useful in practice? A comparison with rapid scoring functions for the p38 MAP kinase protein system.. J Med Chem.

[20] Kollman P (1993). Free-Energy Calculations - Applications to Chemical and Biochemical Phenomena.. Chemical Reviews.

[21] Aqvist J, Luzhkov VB, Brandsdal BO (2002). Ligand binding affinities from MD simulations.. Acc Chem Res.

[22] Kollman PA, Massova I, Reyes C, Kuhn B, Huo SH (2000). Calculating structures and free energies of complex molecules: Combining molecular mechanics and continuum models.. Accounts of Chemical Research.

[23] Huo S, Wang J, Cieplak P, Kollman PA, Kuntz ID (2002). Molecular dynamics and free energy analyses of cathepsin D-inhibitor interactions: insight into structure-based ligand design.. J Med Chem.

[24] Masukawa KM, Kollman PA, Kuntz ID (2003). Investigation of neuraminidase-substrate recognition using molecular dynamics and free energy calculations.. J Med Chem.

[25] Kuhn B, Gerber P, Schulz-Gasch T, Stahl M (2005). Validation and use of the MM-PBSA approach for drug discovery.. J Med Chem.

[26] Ferrara P, Curioni A, Vangrevelinghe E, Meyer T, Mordasini T (2006). New scoring functions for virtual screening from molecular dynamics simulations with a quantum-refined force-field (QRFF-MD). Application to cyclin-dependent kinase 2.. J Chem Inf Model.

[27] Narumi T, Ohno Y, Okimoto N, Koishi T, Suenaga A (2006). A 185 Tflops simulation of amyloid-forming peptides from Yeast Prion Sup35 with the special-purpose computer System MD-GRAPE3..

[28] Taiji M (2004). MDGRAPE-3 chip: a 165 Gflops application specific LSI for molecular dynamics simulations.; 2004..

[29] Thomas MP, McInnes C, Fischer PM (2006). Protein structures in virtual screening: A case study with CDK2.. J Med Chem.

[30] Kontoyianni M, McClellan LM, Sokol GS (2004). Evaluation of docking performance: comparative data on docking algorithms.. J Med Chem.

[31] Wang R, Lu Y, Wang S (2003). Comparative evaluation of 11 scoring functions for molecular docking.. J Med Chem.

[32] Cho AE, Wendel JA, Vaidehi N, Kekenes-Huskey PM, Floriano WB (2005). The MPSim-Dock hierarchical docking algorithm: application to the eight trypsin inhibitor cocrystals.. J Comput Chem.

[33] Erickson JA, Jalaie M, Robertson DH, Lewis RA, Vieth M (2004). Lessons in molecular recognition: the effects of ligand and protein flexibility on molecular docking accuracy.. J Med Chem.

[34] Kua J, Zhang Y, McCammon JA (2002). Studying enzyme binding specificity in acetylcholinesterase using a combined molecular dynamics and multiple docking approach.. J Am Chem Soc.

[35] Witten IH, Frank E (1999). Data mining: practical machine learning tools and techniques with java implementations.

[36] Habe H, Morii K, Fushinobu S, Nam JW, Ayabe Y (2003). Crystal structure of a histidine-tagged serine hydrolase involved in the carbazole degradation (CarC enzyme).. Biochem Biophys Res Commun.

[37] Dorfman DD, Berbaum KS, Metz CE (1992). Receiver operating characteristic rating analysis. Generalization to the population of readers and patients with the jackknife method.. Invest Radiol.

[38] Hillis SL, Berbaum KS (2005). Monte Carlo validation of the Dorfman-Berbaum-Metz method using normalized pseudovalues and less data-based model simplification.. Acad Radiol.

[39] Hillis SL, Obuchowski NA, Schartz KM, Berbaum KS (2005). A comparison of the Dorfman-Berbaum-Metz and Obuchowski-Rockette methods for receiver operating characteristic (ROC) data.. Stat Med.

[40] Roe CA, Metz CE (1997). Variance-component modeling in the analysis of receiver operating characteristic index estimates.. Acad Radiol.

[41] Roe CA, Metz CE (1997). Dorfman-Berbaum-Metz method for statistical analysis of multireader, multimodality receiver operating characteristic data: validation with computer simulation.. Acad Radiol.

[42] Gohlke H, Case DA (2004). Converging free energy estimates: MM-PB(GB)SA studies on the protein-protein complex Ras-Raf.. Journal of Computational Chemistry.

[43] Numata J, Wan M, Knapp EW (2007). Conformational entropy of biomolecules: beyond the quasi-harmonic approximation.. Genome Inform.

[44] Chang CE, Chen W, Gilson MK (2005). Evaluating the accuracy of the quasiharmonic approximation.. Journal of Chemical Theory and Computation.

[45] Davies TG, Bentley J, Arris CE, Boyle FT, Curtin NJ (2002). Structure-based design of a potent purine-based cyclin-dependent kinase inhibitor.. Nat Struct Biol.

[46] Davis ST, Benson BG, Bramson HN, Chapman DE, Dickerson SH (2001). Prevention of chemotherapy-induced alopecia in rats by CDK inhibitors.. Science.

[47] Lamb ML, Jorgensen WL (1997). Computational approaches to molecular recognition.. Curr Opin Chem Biol.

[48] Zhou Z, Madura JD (2004). Relative free energy of binding and binding mode calculations of HIV-1 RT inhibitors based on dock-MM-PB/GS.. Proteins.

[49] Huang N, Shoichet BK, Irwin JJ (2006). Benchmarking sets for molecular docking.. J Med Chem.

[50] Kollman PA, Massova I, Reyes C, Kuhn B, Huo S (2000). Calculating structures and free energies of complex molecules: combining molecular mechanics and continuum models.. Acc Chem Res.

[51] Katz BA, Mackman R, Luong C, Radika K, Martelli A (2000). Structural basis for selectivity of a small molecule, S1-binding, submicromolar inhibitor of urokinase-type plasminogen activator.. Chem Biol.

[52] Ala PJ, DeLoskey RJ, Huston EE, Jadhav PK, Lam PY (1998). Molecular recognition of cyclic urea HIV-1 protease inhibitors.. J Biol Chem.

[53] Dvir H, Wong DM, Harel M, Barril X, Orozco M (2002). 3D structure of Torpedo californica acetylcholinesterase complexed with huprine X at 2.1 A resolution: kinetic and molecular dynamic correlates.. Biochemistry.

[54] MOE

[55] Lipinski CA, Lombardo F, Dominy BW, Feeney PJ (2001). Experimental and computational approaches to estimate solubility and permeability in drug discovery and development settings.. Adv Drug Deliv Rev.

[56] Wang R, Fang X, Lu Y, Yang CY, Wang S (2005). The PDBbind database: methodologies and updates.. J Med Chem.

[57] Wang R, Fang X, Lu Y, Wang S (2004). The PDBbind database: collection of binding affinities for protein-ligand complexes with known three-dimensional structures.. J Med Chem.

[58] Gray NS, Wodicka L, Thunnissen AMWH, Norman TC, Kwon SJ (1998). Exploiting chemical libraries, structure, and genomics in the search for kinase inhibitors.. Science.

[59] Schrödinger Inc

[60] Sørenson T (1948). A method of establishing groups of equal amplitude in a plant based on similarity of species content and its applications to analysis of vegetation on Danish commons.. Biologiske Skrifter.

[61] Hoffmann D, Kramer B, Washio T, Steinmetzer T, Rarey M (1999). Two-stage method for protein-ligand docking.. J Med Chem.

[62] Jorgensen WL, Chandrasekhar J, Madura JD, Impey RW, Klein ML (1983). Comparison of Simple Potential Functions for Simulating Liquid Water.. Journal of Chemical Physics.

[63] Case DA, Darden TA, Cheatham TEr, Simmerling CL, Wang J (2004). AMBER 8.

[64] Duan Y, Wu C, Chowdhury S, Lee MC, Xiong G (2003). A point-charge force field for molecular mechanics simulations of proteins based on condensed-phase quantum mechanical calculations.. J Comput Chem.

[65] Berendsen HJC, Postma JMP, van Gunsteren WF, DiNola A, Haak JR (1984). Molecular dynamics with coupling to an external bath.. J Comput Phys.

[66] Wang J, Wolf RM, Caldwell JW, Kollman PA, Case DA (2004). Development and testing of a general amber force field.. J Comput Chem.

[67] Jakalian A, Jack DB, Bayly CI (2002). Fast, efficient generation of high-quality atomic charges. AM1-BCC model: II. Parameterization and validation.. J Comput Chem.

[68] Jakalian A, Bush BL, Jack DB, Bayly CI (2000). Fast, efficient generation of high-quality atomic Charges. AM1-BCC model: I. Method.. Journal of Computational Chemistry.

[69] Rocchia W, Alexov E, Honig B (2001). Extending the applicability of the nonlinear poisson-boltzmann equation: multiple dielectric constants and multivalent ions.. J Phys Chem B.

[70] Rocchia W, Sridharan S, Nicholls A, Alexov E, Chiabrera A (2002). Rapid grid-based construction of the molecular surface and the use of induced surface charge to calculate reaction field energies: applications to the molecular systems and geometric objects.. J Comput Chem.

[71] Sitkoff D, Sharp KA, Honig B (1994). Accurate calculation of hydration free energies using macroscopic solvent models.. J Phys Chem.

[72] Swanson JMJ, Adcock SA, McCammon JA (2005). Optimized radii for Poisson-Boltzmann calculations with the AMBER force field.. Journal of Chemical Theory and Computation.

[73] Sanner MF, Olson AJ, Spehner JC (1996). Reduced surface: an efficient way to compute molecular surfaces.. Biopolymers.

[74] Levy RM, Karplus M, Kushick J, Perahia D (1984). Evaluation of the Configurational Entropy for Proteins - Application to Molecular-Dynamics Simulations of an Alpha-Helix.. Macromolecules.

