# Supplementary material for: High-Performance Drug Discovery: Computational Screening by Combining Docking and Molecular Dynamics Simulations
Source: PLoS Comput Biol. 2009 Oct 9;5(10):e1000528. doi: 10.1371/journal.pcbi.1000528 (PMC2746282; doi:10.1371/journal.pcbi.1000528)
Supplement: Figure S3 — Active compounds of AChE. The structural formulae and PDB ids of active compounds used in the seeded compound library are shown in the following figures. The asterisks represent the active compounds in top-scoring 1,000. (0.06 MB DOC) [file pcbi.1000528.s003.doc]

**Figure S3. Active compounds of AChE.**

The structural formulae and PDB ids of active compounds used in the seeded compound library are shown in the following figures. The asterisks represent the active compounds in top-scoring 1,000.

**
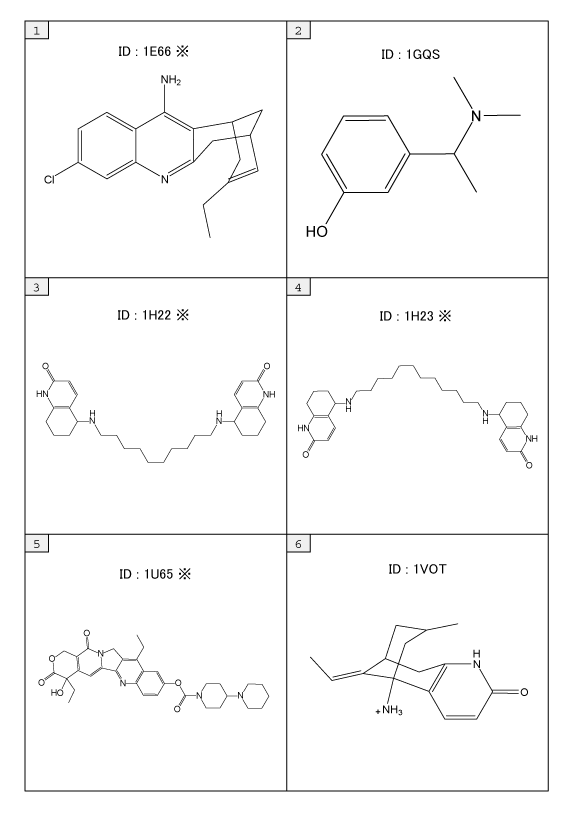

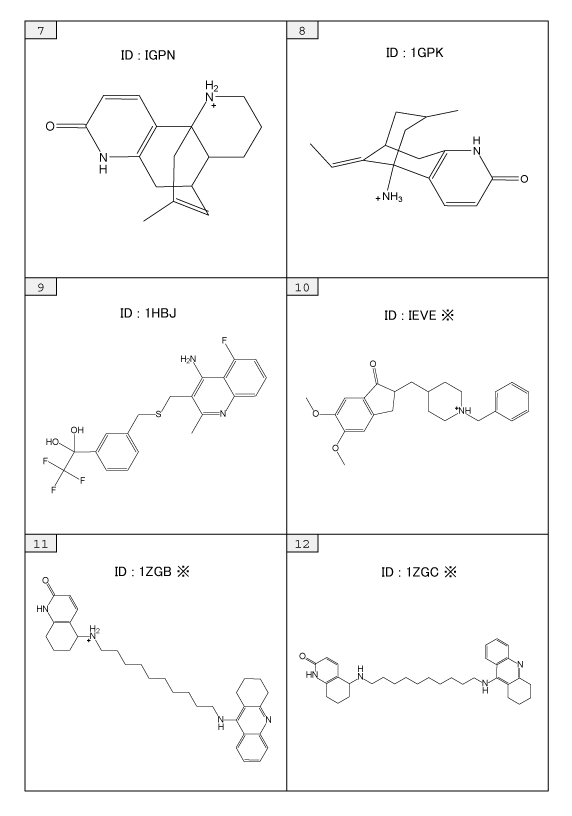

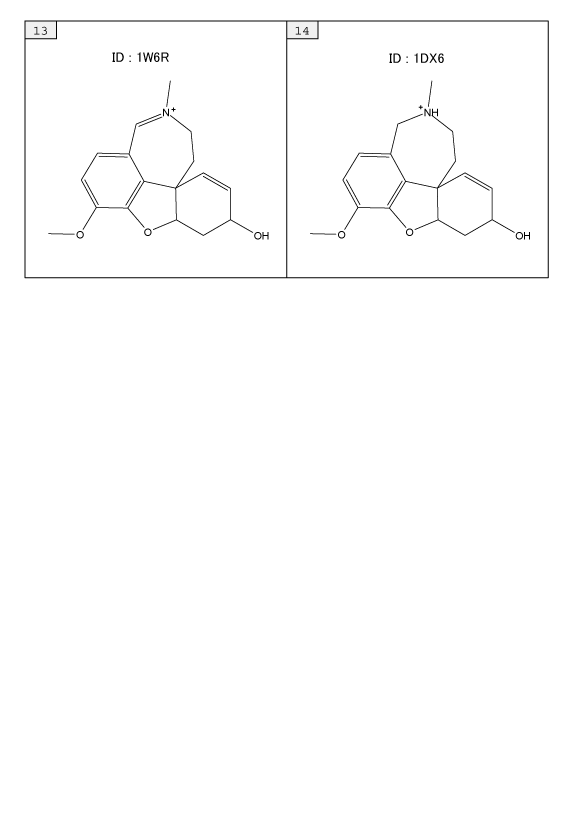
**
