# Supplementary material for: High-Performance Drug Discovery: Computational Screening by Combining Docking and Molecular Dynamics Simulations
Source: PLoS Comput Biol. 2009 Oct 9;5(10):e1000528. doi: 10.1371/journal.pcbi.1000528 (PMC2746282; doi:10.1371/journal.pcbi.1000528)
Supplement: Figure S4 — Active compounds of CDK2. The structural formulae and PDB ids of active compounds used in the seeded compound library are shown in the following figures. The asterisks represent the active compounds in top-scoring 1,000. Compounds 1 and 19 were selected by referencing literatures. (0.11 MB DOC) [file pcbi.1000528.s004.doc]

**Figure S4. Active compounds of CDK2.**

The structural formulae and PDB ids of active compounds used in the seeded compound library are shown in the following figures. The asterisks represent the active compounds in top-scoring 1,000. Compounds 1 and 19 were selected by referencing literatures.[1,2]


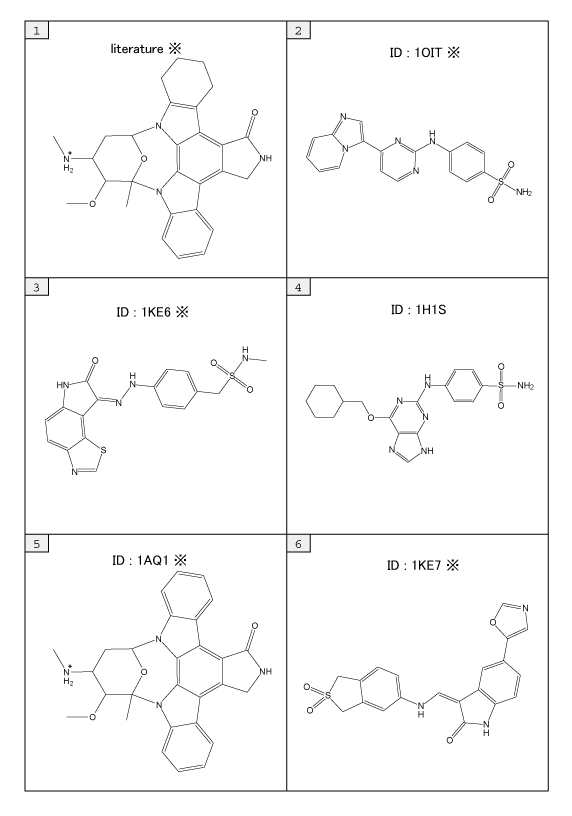

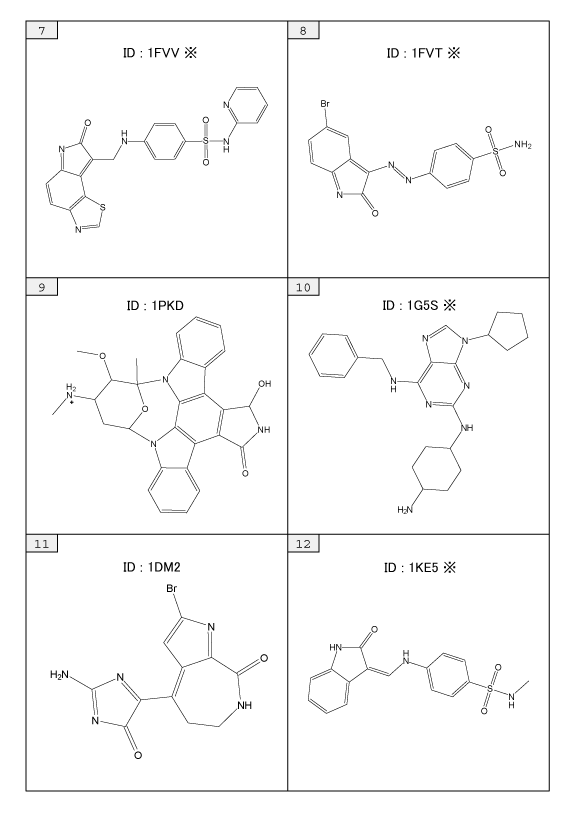

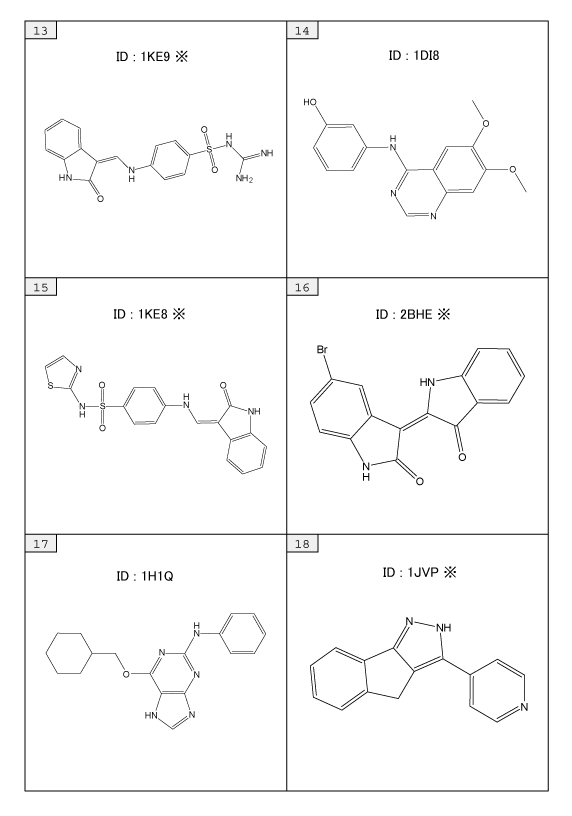

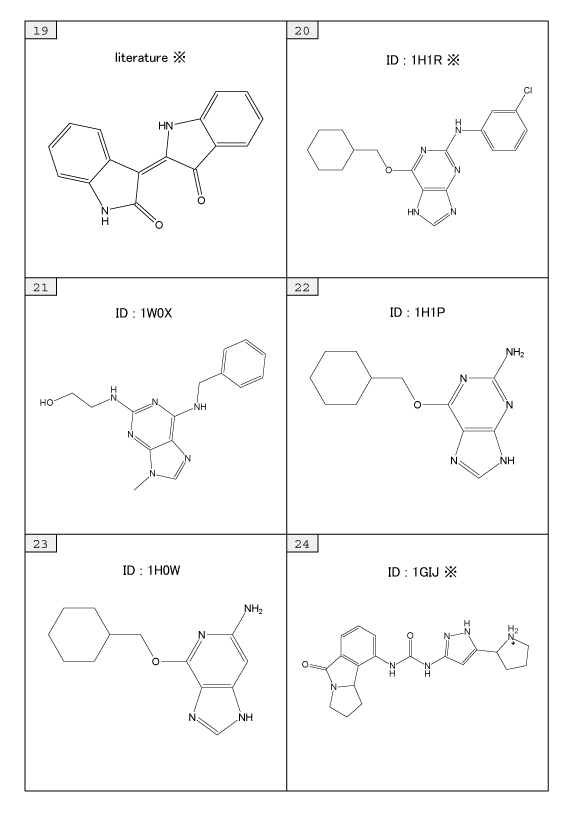

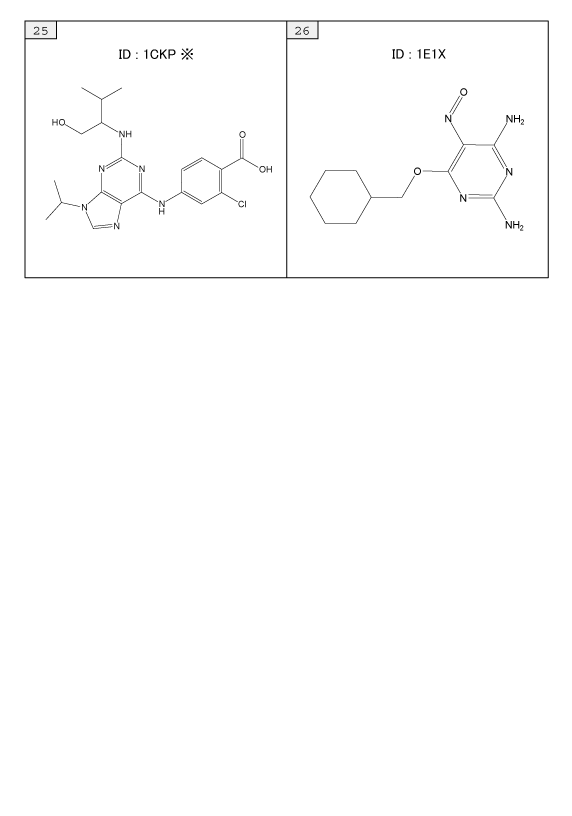


**References**

1. Ferrara P, Curioni A, Vangrevelinghe E, Meyer T, Mordasini T, et al. (2006) New scoring functions for virtual screening from molecular dynamics simulations with a quantum-refined force-field (QRFF-MD). Application to cyclin-dependent kinase 2. J Chem Inf Model 46: 254-263.

2. Gray NS, Wodicka L, Thunnissen AMWH, Norman TC, Kwon SJ, et al. (1998) Exploiting chemical libraries, structure, and genomics in the search for kinase inhibitors. Science 281: 533-538.
