# Supplementary material for: High-Performance Drug Discovery: Computational Screening by Combining Docking and Molecular Dynamics Simulations
Source: PLoS Comput Biol. 2009 Oct 9;5(10):e1000528. doi: 10.1371/journal.pcbi.1000528 (PMC2746282; doi:10.1371/journal.pcbi.1000528)

**Figure S5. Number of correctly docked conformations in top-scored active compounds.**

These indicate the number of correctly docked conformations in the top-scoring poses for active compounds obtained from molecular docking, G01, and G06. The red and blue bars indicate the number of poses within the root mean square deviations (RMSDs) of 2.5 and 3.5 Å from those of the experimental structure, respectively. The active compounds in the top 1,000 were investigated. In G06, the final MD structure was used.


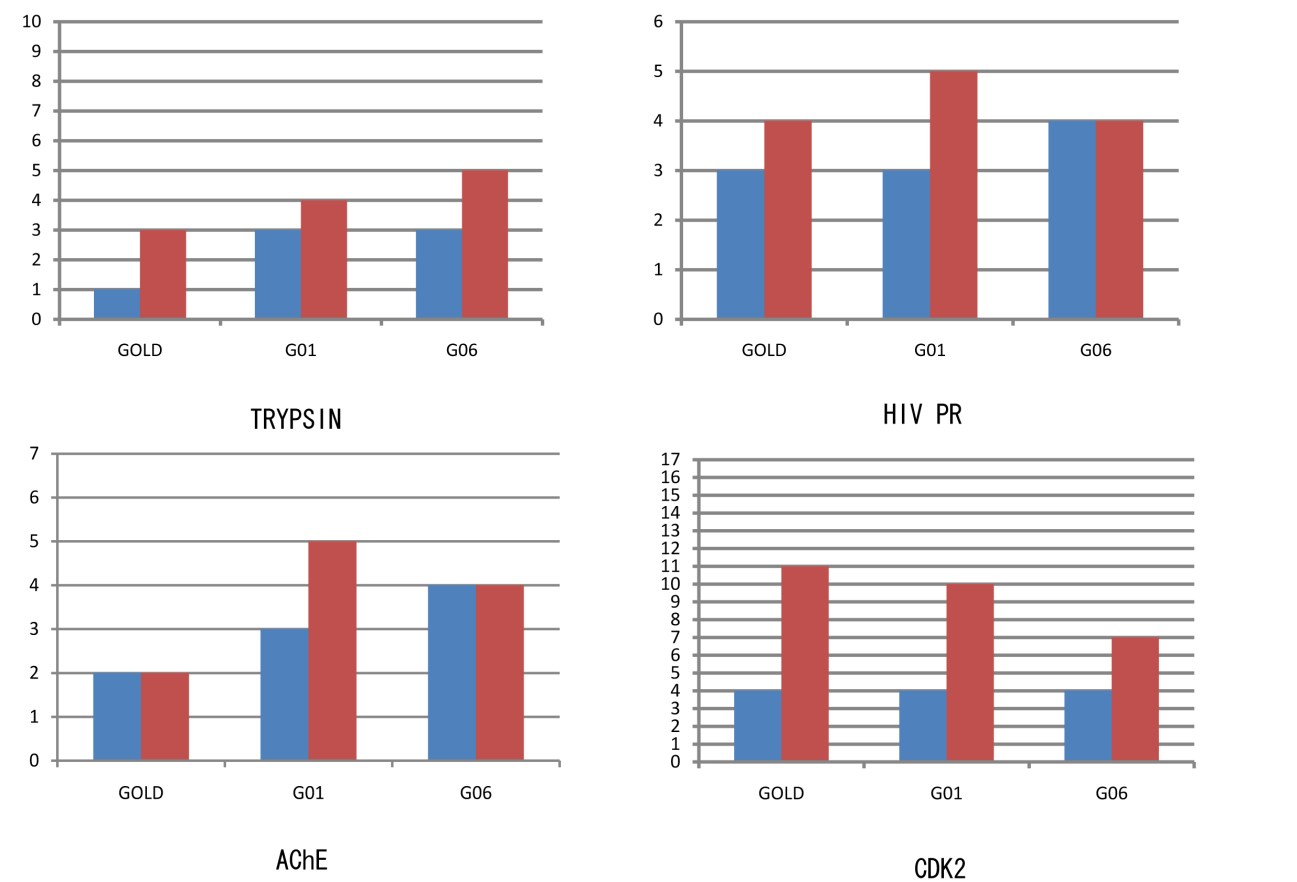

Supplement: Figure S5 — Number of correctly docked conformations in top-scored active compounds. These indicate the number of correctly docked conformations in the top-scoring poses for active compounds obtained from molecular docking, G01, and G06. The red and blue bars indicate the number of poses within the root mean square deviations (RMSDs) of 2.5 and 3.5 Å from those of the experimental structure, respectively. The active compounds in the top 1,000 were investigated. In G06, the final MD structure was used. (0.10 MB DOC) [file pcbi.1000528.s005.doc]
