# Supplementary material for: High-Performance Drug Discovery: Computational Screening by Combining Docking and Molecular Dynamics Simulations
Source: PLoS Comput Biol. 2009 Oct 9;5(10):e1000528. doi: 10.1371/journal.pcbi.1000528 (PMC2746282; doi:10.1371/journal.pcbi.1000528)

**Figure S6. Minimal RMSD values of computed poses from experimental poses for active compounds.**

The horizontal axis indicates the index number of active compounds in the top 1,000 shown in Figures S1–S4 and the vertical axis indicates the minimal RMSD among all the poses. For each protein, the poses obtained from molecular docking, G01, and G06 were investigated. In G06, the final MD structure was used. The red bars indicate the pose within the top-three scoring. For trypsin, HIV PR, and AChE, it was found that MD simulations could improve the binding modes and predict better binding free energies. For CDK2, however, it is suggested that MD simulations lead to structural uncertainties and an inaccurate estimation of the binding free energy.

**Trypsin**


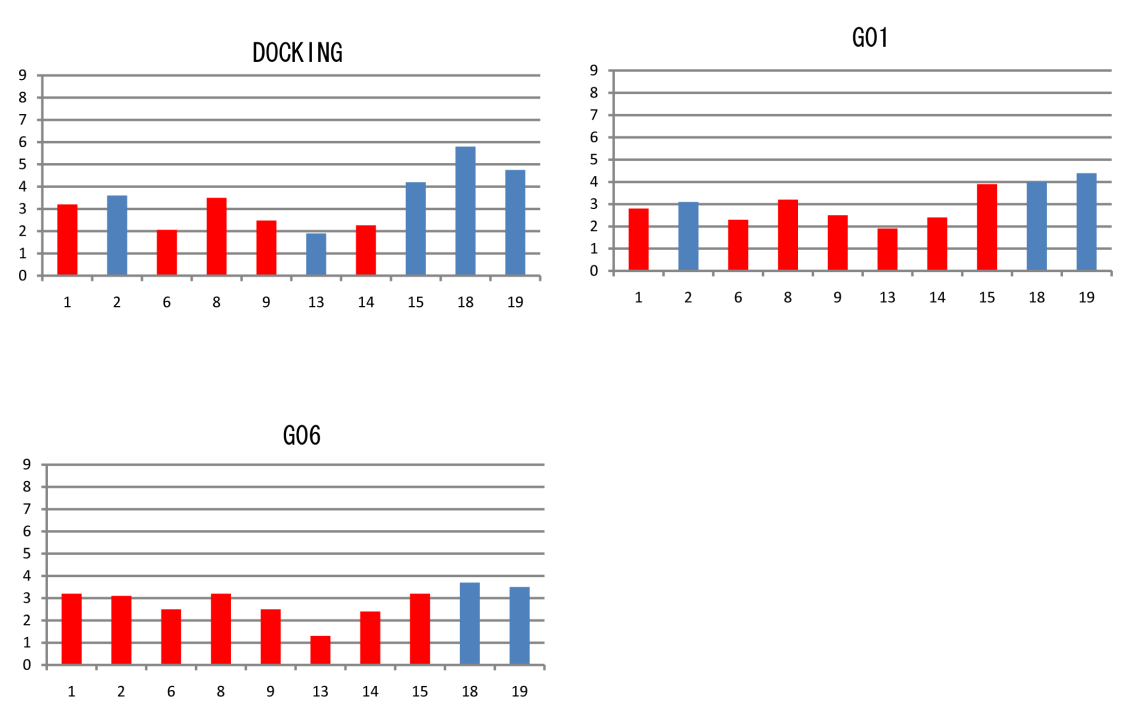


**HIV PR**


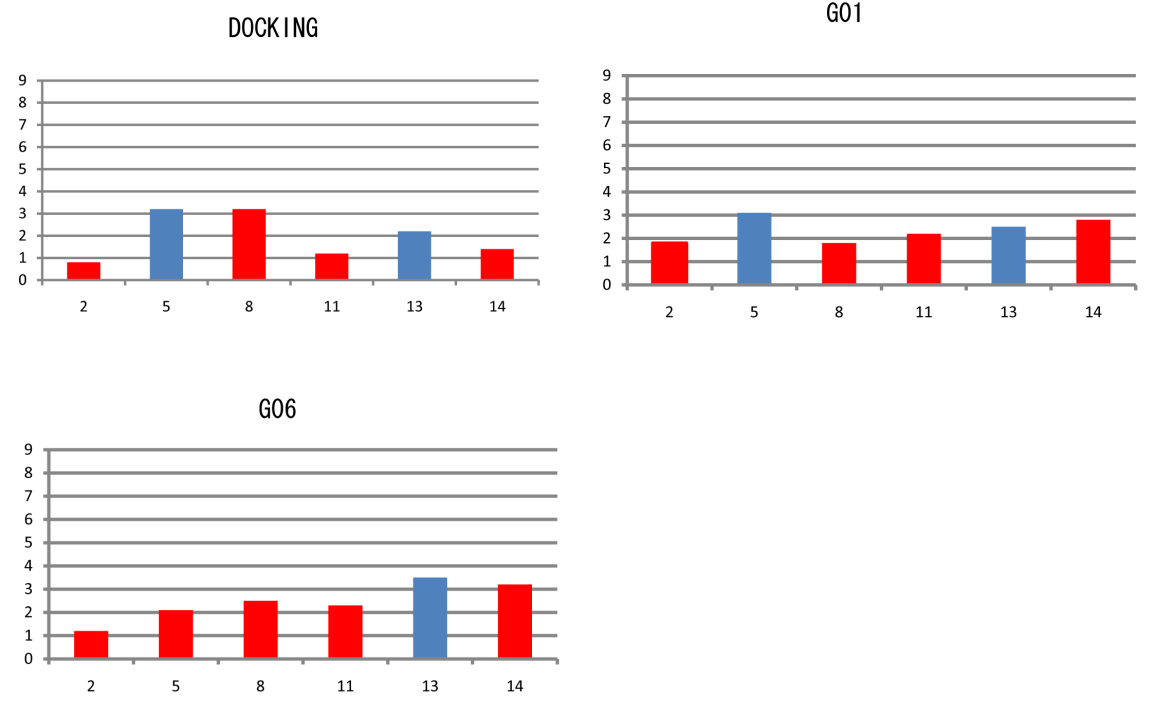


**AChE**


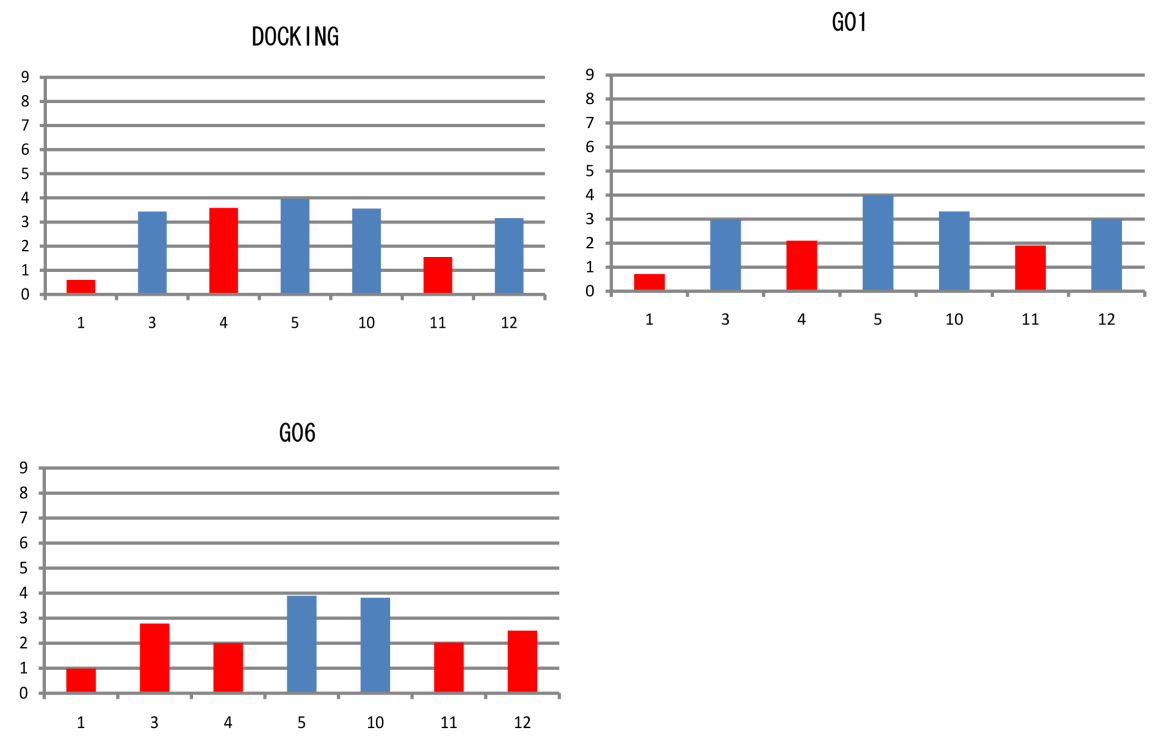


**CDK2**


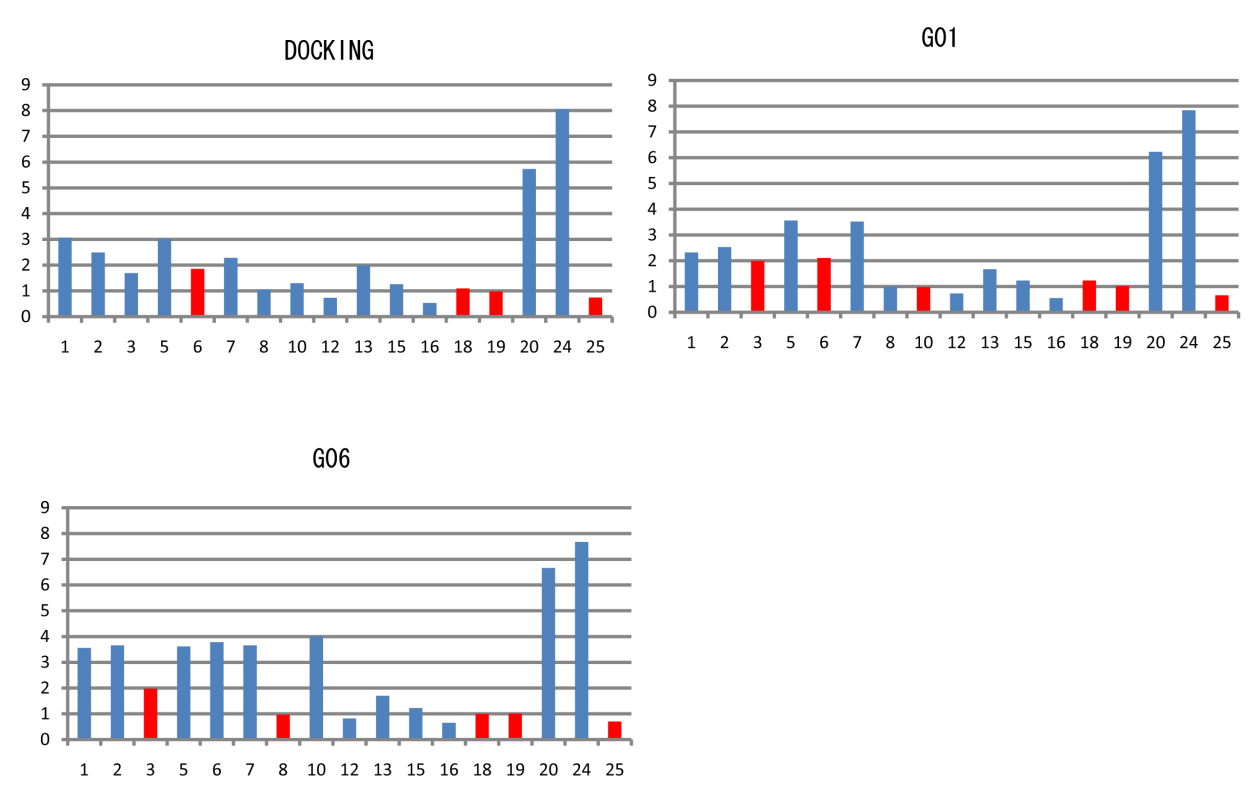

Supplement: Figure S6 — Minimal RMSD values of computed poses from experimental poses for active compounds. The horizontal axis indicates the index number of active compounds in the top 1,000 shown in Figures S1, S2, S3, S4 and the vertical axis indicates the minimal RMSD among all the poses. For each protein, the poses obtained from molecular docking, G01, and G06 were investigated. In G06, the final MD structure was used. The red bars indicate the pose within the top-three scoring. For trypsin, HIV PR, and AChE, it was found that MD simulations could improve the binding modes and predict better binding free energies. For CDK2, however, it is suggested that MD simulations lead to structural uncertainties and an inaccurate estimation of the binding free energy. (0.24 MB DOC) [file pcbi.1000528.s006.doc]
