# Supplementary material for: High-Performance Drug Discovery: Computational Screening by Combining Docking and Molecular Dynamics Simulations
Source: PLoS Comput Biol. 2009 Oct 9;5(10):e1000528. doi: 10.1371/journal.pcbi.1000528 (PMC2746282; doi:10.1371/journal.pcbi.1000528)

**Figure S7. ROC curves using molecular weight as classifier.**

This graph shows the sensitivity versus 1-specificity. This indicates ROC curves when the active compounds in the top 1,000 compounds are considered as total the true positives. ROC curves for trypsin, HIV PR, AChE, and CDK2 were drawn in blue, red, yellow, and orange, respectively. These ROC values for trypsin, HIV PR, AChE, and CDK2 are 0.454, 0.674, 0.462, and 0.430. From statistical analysis, it is obvious that the differences in the ROC values between G06 and molecular weight were statistically significant for trypsin, HIV PR, AChE. The differences in the ROC values between molecular docking and molecular weight were not statistically significant for all proteins.


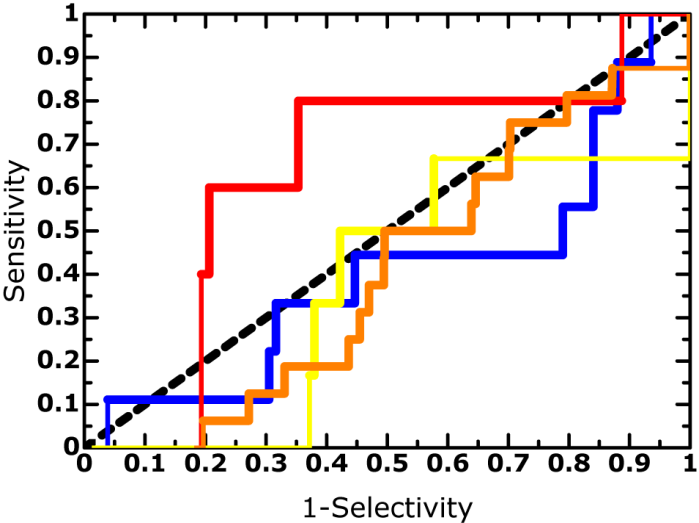

Supplement: Figure S7 — ROC curves using molecular weight as classifier. This graph shows the sensitivity versus 1-specificity. This indicates ROC curves when the active compounds in the top 1,000 compounds are considered as total the true positives. ROC curves for trypsin, HIV PR, AChE, and CDK2 were drawn in blue, red, yellow, and orange, respectively. These ROC values for trypsin, HIV PR, AChE, and CDK2 are 0.454, 0.674, 0.462, and 0.430. From statistical analysis, it is obvious that the differences in the ROC values between G06 and molecular weight were statistically significant for trypsin, HIV PR, AChE. The differences in the ROC values between molecular docking and molecular weight were not statistically significant for all proteins. (0.08 MB DOC) [file pcbi.1000528.s007.doc]
