# Supplementary material for: High-Performance Drug Discovery: Computational Screening by Combining Docking and Molecular Dynamics Simulations
Source: PLoS Comput Biol. 2009 Oct 9;5(10):e1000528. doi: 10.1371/journal.pcbi.1000528 (PMC2746282; doi:10.1371/journal.pcbi.1000528)
Supplement: Text S1 — Assignment of missing force field parameters. We filled in the following missing parameters on the basis of the information on regarding atom types, bonds, valences, angles, and dihedrals by using an in-house program. (0.06 MB DOC) [file pcbi.1000528.s008.doc]

**TEXT S1. Assignment of missing force field parameters.**

We filled in the following missing parameters on the basis of the information on regarding atom types, bonds, valences, angles, and dihedrals by using an in-house program.

ne-cc 381.8 1.414 SOURCE3 7 0.0093 0.0103

ce-cc 519.2 1.362 SOURCE3 1 same as cc-cf

sy-cd 248.9 1.782 SOURCE3 5 0.0105 0.0114

cx-ce 327.3 1.509 SOURCE1 20 0.0000 0.0000

cc-br 278.7 1.883 SOURCE1 31 0.0000 0.0000

sy-n3 251.3 1.761 SOURCE3 6 0.0563 0.0766

cd-nh-ca 65.5 123.66 SOURCE3 3 0.9054 0.9603

os-ca-nb 60.0 120.00 SOURCE0 1

oh-c2-ce 71.6 122.28 SOURCE3 4 0.5600 0.5600

os-n2-ce 72.0 110.01 SOURCE3 5 3.1049 4.2241

cp-ca-c 67.2 119.07 SOURCE3 14 2.0133 2.3077

hx-c3-cc 47.4 108.64 SOURCE3 1 0.0000 0.0000

sy-nh-c2 64.0 117.74 SOURCE3 1 0.0000 0.0000

nb-ca-ce 66.9 126.21 SOURCE3 1 same as cd-ca-nb

nh-ce-n2 73.9 111.94 SOURCE3 1 same as nd-cd-nh

cg-ne-c2 60.0 120.00 SOURCE0 1

nh-c3-cd 60.0 109.47 SOURCE0 1

h2-c3-ca 60.0 109.47 SOURCE0 1

cc-ce-c3 64.7 115.97 SOURCE3 4 2.7731 3.0507

n4-c3-ca 65.8 111.54 SOURCE3 1 0.0000 0.0000

s6-c3-ca 60.0 109.47 SOURCE0 1

n3-c3-cd 66.5 111.47 SOURCE3 3 2.1065 2.2409

n -c3-ca 66.7 111.56 SOURCE3 28 0.7771 1.7981

n4-c3-cc 65.8 111.54 SOURCE3 1 0.0000 0.0000

ss-c3-hx 60.0 109.47 SOURCE0 1

ne-cc-ca 69.7 118.32 SOURCE3 7 0.8179 1.0468

na-cc-c 72.2 106.80 SOURCE3 33 0.4847 0.6297

n2-ce-cx 68.6 116.94 SOURCE3 2 0.0000 0.0000

oh-c3-n 69.2 119.75 SOURCE3 2 0.0000 0.0000

nh-n2-cc 70.5 119.08 SOURCE3 2 0.0000 0.0000

oh-cd-cd 68.8 126.44 SOURCE3 3 0.5018 0.5018

sy-cd-cc 62.0 120.35 SOURCE3 5 0.4293 0.5401

nh-ce-nh 72.9 115.96 SOURCE3 1 0.0000 0.0000

nc-c -cc 70.0 113.42 SOURCE3 4 1.1985 1.3957

cx-cx-ce 72.6 88.75 SOURCE2 2 31.0500 31.0500

ha-ce-cc 49.3 119.16 SOURCE3 1 same as cc-cf-ha

oh-c3-cc 68.3 109.72 SOURCE3 2 1.8365 1.8365

hc-cx-c3 45.8 117.92 SOURCE3 92 0.7174 1.1927

n -c3-c2 66.7 111.56 SOURCE3 28 0.7771 1.7981

cf-ce-cc 63.8 130.92 SOURCE3 2 same as ce-cf-cf

cd-ce-cc 63.9 126.07 SOURCE3 1 same as c -cf-cc

hc-cx-cu 44.5 124.20 SOURCE2 2 0.0000 0.0000

ce-c3-c3 64.7 108.10 SOURCE3 5 4.5525 4.8691

nd-cc-ce 71.1 112.56 SOURCE3 141 3.3765 4.2871

n -cc-c3 67.9 115.15 SOURCE3 153 1.9677 2.7443

hx-c3-ca 47.4 108.64 SOURCE3 1 0.0000 0.0000

ss-c3-n4 60.0 109.47 SOURCE0 1

o -sy-cd 66.4 107.25 SOURCE3 10 0.5364 0.5477

n2-cc-ca 69.3 121.15 SOURCE3 6 0.4642 0.4642

ss-c3-ca 63.6 104.97 SOURCE3 2 2.2248 2.2248

n -cd-c3 67.9 115.15 SOURCE3 153 1.9677 2.7443

na-c3-ca 68.6 105.72 SOURCE3 2 2.6464 2.6464

sy-nh-cc 64.0 117.74 SOURCE3 1 0.0000 0.0000

ca-c3-c2 65.2 108.08 SOURCE3 8 3.1217 4.1083

ce-cc-c 65.6 123.92 SOURCE3 1 same as cc-cc-cf

ss-cc-ce 63.8 115.02 SOURCE3 2 0.0000 0.0000

na-ca-cp 70.2 118.34 SOURCE3 54 2.1861 3.6168

hx-c3-cd 47.4 108.64 SOURCE3 1 0.0000 0.0000

cd-c3-c2 67.3 102.35 SOURCE3 1 0.0000 0.0000

ce-c3-ca 65.2 108.08 SOURCE3 8 3.1217 4.1083

ha-cu-cx 45.1 121.52 SOURCE3 32 2.8716 3.2091

nh-c2-cc 69.8 124.99 SOURCE3 7 0.8702 0.9929

sy-n3-hn 42.5 108.93 SOURCE3 7 1.5575 1.7819

f -c3-ca 66.4 111.30 SOURCE2 2 0.4000 0.4000

cc-c3-c 65.5 107.99 SOURCE3 10 5.0855 5.1937

n4-c3-c2 65.8 111.54 SOURCE3 1 0.0000 0.0000

n3-c3-cc 66.5 111.47 SOURCE3 3 2.1065 2.2409

cx-ce-ca 101.0 50.80 SOURCE2 1 0.0000 0.0000

n3-c3-ca 66.5 111.47 SOURCE3 3 2.1065 2.2409

nh-c3-ca 60.0 109.47 SOURCE0 1

cu-cx-c3 64.7 108.10 SOURCE3 5 4.5525 4.8691

ce-nh-c3 63.7 121.18 SOURCE3 1 0.0000 0.0000

nh-c3-cc 60.0 109.47 SOURCE0 1

os-c -ce 70.5 112.30 SOURCE3 6 2.3802 2.7842

ce-cx-ca 65.2 108.08 SOURCE3 8 3.1217 4.1083

na-c2-cf 69.8 121.38 SOURCE3 26 5.2386 6.9463

cd-ce-c3 64.8 119.45 SOURCE3 35 7.1944 8.2040

n4-c3-cd 65.8 111.54 SOURCE3 1 0.0000 0.0000

nf-ne-cc 69.9 114.72 SOURCE3 2 0.0000 0.0000

ce-cc-cd 65.6 123.92 SOURCE3 1 same as cc-cc-cf

n3-sy-ca 60.0 109.47 SOURCE0 1

sy-n3-c3 61.4 112.91 SOURCE3 3 0.8469 0.8983

oh-c3-n3 60.0 109.47 SOURCE0 1

nh-ce-cc 66.7 122.76 SOURCE3 1 0.0000 0.0000

nh-ce-ca 66.7 122.76 SOURCE3 1 0.0000 0.0000

na-cc-ca 72.2 106.80 SOURCE3 33 0.4847 0.6297

oh-c3-h3 60.0 109.47 SOURCE0 1

o -sy-n3 67.6 109.07 SOURCE3 6 1.7576 2.3605

na-cc-ce 72.2 106.80 SOURCE3 33 0.4847 0.6297

cx-c3-cc 72.6 88.75 SOURCE2 2 31.0500 31.0500

ss-cc-nh 60.0 120.00 SOURCE0 1

cd-cc-br 63.1 122.42 SOURCE3 3 0.0663 0.0703

cx-c3-ca 72.6 88.75 SOURCE2 2 31.0500 31.0500

sy-ca-nb 60.0 120.00 SOURCE0 1

nc-cc-br 60.0 120.00 SOURCE0 1

ne-cc-c 69.7 118.32 SOURCE3 7 0.8179 1.0468

n3-sy-cd 60.0 109.47 SOURCE0 1

na-cd-c 72.9 109.42 SOURCE3 265 1.3410 2.6051

nb-ca-c3 60.0 120.00 SOURCE0 1

sy-cd-ss 60.0 120.00 SOURCE0 1

cx-cx-ca 72.6 88.75 SOURCE2 2 31.0500 31.0500

ss-cd-sy-o 6 7.600 180.000 2.000

nc-cd-ca-ca 4 16.000 180.000 2.000 statistic value of parm94

ha-cu-cx-cu 6 0.000 0.000 3.000 same as X-c3-ca-X

ca-c3-ce-c 6 0.000 0.000 3.000 same as X-c3-ca-X

nf-ne-cc-c 2 1.600 180.000 2.000 single bond

ha-cu-cu-ha 4 8.700 180.000 2.000 intrpol.bsd.on C6H6

ha-cu-cx-c3 6 0.000 0.000 3.000 same as X-c3-ca-X

cd-cc-os-c 2 2.100 180.000 2.000 parm99

cc-ce-c3-c3 6 0.000 0.000 3.000 same as X-c3-ca-X

nd-cc-ss-ca 2 2.200 180.000 2.000

nb-ca-cc-cd 4 14.500 180.000 2.000 intrpol.bsd.on C6H6

nh-c2-cc-ca 4 26.600 180.000 2.000 c2=c2 double bond, intrpol.bsd.on C6H6

nd-cc-os-cc 2 2.100 180.000 2.000 parm99

na-cc-ca-ca 4 16.000 180.000 2.000 statistic value of parm94

cd-ce-cc-c 4 4.000 180.000 2.000 c2-c2 single bond, parm99

nd-cc-ce-c3 4 16.000 180.000 2.000 statistic value of parm94

ca-ca-cc-c 4 14.500 180.000 2.000 intrpol.bsd.on C6H6

nb-ca-ce-n2 4 14.500 180.000 2.000 intrpol.bsd.on C6H6

ha-ce-ca-ca 4 4.000 180.000 2.000 c2-c2 single bond, parm99

cd-cc-ca-ca 4 16.000 180.000 2.000 statistic value of parm94

ss-cd-sy-n3 6 7.600 180.000 2.000

ca-ce-nh-c3 4 4.200 180.000 2.000 same as X-ca-nh-X

ca-ca-cc-c3 4 14.500 180.000 2.000 intrpol.bsd.on C6H6

h5-cd-ss-ca 2 2.200 180.000 2.000

cd-ce-c3-c3 6 0.000 0.000 3.000 same as X-c3-ca-X

nh-n2-cc-c 2 8.300 180.000 2.000 double bond, parm99

hc-c3-ce-c2 6 0.000 0.000 3.000 same as X-c3-ca-X

ha-cd-ca-ca 4 16.000 180.000 2.000 statistic value of parm94

nd-cc-ca-ca 4 16.000 180.000 2.000 statistic value of parm94

c3-c3-ce-c2 6 0.000 0.000 3.000 same as X-c3-ca-X

nh-n2-cc-ca 2 8.300 180.000 2.000 double bond, parm99

c3-cc-os-c 2 2.100 180.000 2.000 parm99

nb-ca-cc-na 4 14.500 180.000 2.000 intrpol.bsd.on C6H6

cx-ce-ca-ca 4 4.000 180.000 2.000 c2-c2 single bond, parm99

hc-cx-c3-ca 9 1.400 0.000 3.000 same as X-c3-c3-X

nc-cd-ss-ca 2 2.200 180.000 2.000

nf-ne-cc-ca 2 1.600 180.000 2.000 single bond

nb-ca-cd-cc 4 14.500 180.000 2.000 intrpol.bsd.on C6H6

cd-cc-ss-cc 2 2.200 180.000 2.000

cx-cx-c3-cc 9 1.400 0.000 3.000 same as X-c3-c3-X

nd-cc-ce-cf 4 16.000 180.000 2.000 statistic value of parm94

n2-ce-ca-ca 4 4.000 180.000 2.000 c2-c2 single bond, parm99

n2-cc-ca-ca 4 16.000 180.000 2.000 statistic value of parm94

n2-ce-cx-ca 6 0.000 0.000 3.000 same as X-c3-ca-X

hn-n2-ce-ca 2 8.300 180.000 2.000 double bond, parm99

c3-ce-cc-c 4 4.000 180.000 2.000 c2-c2 single bond, parm99

ha-ce-nh-c3 4 4.200 180.000 2.000 same as X-ca-nh-X

hn-nh-ce-ca 4 4.200 180.000 2.000 same as X-ca-nh-X

nd-cc-ss-cc 2 2.200 180.000 2.000

n3-sy-cd-cc 6 7.600 180.000 2.000

nd-cc-ce-ha 4 16.000 180.000 2.000 statistic value of parm94

os-cc-ca-ca 4 16.000 180.000 2.000 statistic value of parm94

hc-cx-c3-hc 9 1.400 0.000 3.000 same as X-c3-c3-X

ce-cc-ss-ca 2 2.200 180.000 2.000

ss-cc-ce-nh 4 16.000 180.000 2.000 statistic value of parm94

na-cc-ce-cf 4 16.000 180.000 2.000 statistic value of parm94

nh-ce-nh-hn 4 4.200 180.000 2.000 same as X-ca-nh-X

hc-cx-cu-cu 6 0.000 180.000 2.000 JCC, 7, (1986), 230

cd-cc-os-cc 2 2.100 180.000 2.000 parm99

ce-cx-ca-ca 6 0.000 180.000 2.000 JCC, 7, (1986), 230

nb-ca-ce-cx 4 14.500 180.000 2.000 intrpol.bsd.on C6H6

nh-ce-cc-cd 4 4.000 180.000 2.000 c2-c2 single bond, parm99

cx-cx-ce-ca 6 0.000 180.000 2.000 JCC, 7, (1986), 230

n1-cg-ne-c2 1 1.100 180.000 1.000

cc-cd-ss-ca 2 2.200 180.000 2.000

h5-cc-ss-ca 2 2.200 180.000 2.000

h5-cc-os-cc 2 2.100 180.000 2.000 parm99

cx-cx-c3-ca 9 1.400 0.000 3.000 same as X-c3-c3-X

hc-c3-cx-cu 9 1.400 0.000 3.000 JCC,7,(1986),230

ho-oh-cd-cd 2 2.100 180.000 2.000 parm99

n -cd-ca-ca 4 16.000 180.000 2.000 statistic value of parm94

cd-cc-ss-ca 2 2.200 180.000 2.000

hc-c3-cx-cx 9 1.400 0.000 3.000 JCC,7,(1986),230

sy-cd-ss-ca 2 2.200 180.000 2.000

cu-cx-c3-cc 9 1.400 0.000 3.000 same as X-c3-c3-X

n2-ce-cx-cx 6 0.000 0.000 3.000 same as X-c3-ca-X

n2-ce-nh-hn 4 4.200 180.000 2.000 same as X-ca-nh-X

ha-cu-cu-cx 4 8.700 180.000 2.000 intrpol.bsd.on C6H6

cx-cx-ca-ca 6 0.000 180.000 2.000 JCC, 7, (1986), 230

ne-cc-ca-ca 4 16.000 180.000 2.000 statistic value of parm94

hn-nh-ce-cc 4 4.200 180.000 2.000 same as X-ca-nh-X

nh-cc-ss-cc 2 2.200 180.000 2.000

cu-cu-cx-c3 6 0.000 0.000 3.000 same as X-c3-ca-X

nh-c2-cc-c 4 26.600 180.000 2.000 c2=c2 double bond, intrpol.bsd.on C6H6

hc-c3-ce-cd 6 0.000 0.000 3.000 same as X-c3-ca-X

nh-ce-ca-ca 4 4.000 180.000 2.000 c2-c2 single bond, parm99

h4-cc-ss-cc 2 2.200 180.000 2.000

ca-c3-ce-c2 6 0.000 0.000 3.000 same as X-c3-ca-X

cd-cd-ca-ca 4 16.000 180.000 2.000 statistic value of parm94

ca-ca-cc-c2 4 14.500 180.000 2.000 intrpol.bsd.on C6H6

ca-ca-cd-c 4 14.500 180.000 2.000 intrpol.bsd.on C6H6

hc-c3-ce-cc 6 0.000 0.000 3.000 same as X-c3-ca-X

cd-cd-ce-c3 4 16.000 180.000 2.000 statistic value of parm94

ha-c2-cc-ca 4 26.600 180.000 2.000 c2=c2 double bond, intrpol.bsd.on C6H6

ha-cc-ca-ca 4 16.000 180.000 2.000 statistic value of parm94

hc-c3-ce-c 6 0.000 0.000 3.000 same as X-c3-ca-X

nh-ce-n2-hn 2 1.600 180.000 2.000 single bond

n1-cg-ca-ca 2 0.000 180.000 2.000 same as X-c-c1-X

os-n2-ce-ca 2 8.300 180.000 2.000 double bond, parm99

cc-os-cc-ca 2 2.100 180.000 2.000 parm99

o -sy-cd-cc 6 7.600 180.000 2.000

ha-c2-cc-c 4 26.600 180.000 2.000 c2=c2 double bond, intrpol.bsd.on C6H6

hc-cx-c3-cc 9 1.400 0.000 3.000 same as X-c3-c3-X

os-n2-ce-cx 2 8.300 180.000 2.000 double bond, parm99

hc-cx-cu-ha 6 0.000 180.000 2.000 JCC, 7, (1986), 230

ho-oh-cd-cc 2 2.100 180.000 2.000 parm99

cd-cd-ce-cc 4 16.000 180.000 2.000 statistic value of parm94

na-cc-ce-ha 4 16.000 180.000 2.000 statistic value of parm94

nd-cc-ce-cd 4 16.000 180.000 2.000 statistic value of parm94

cc-cd-ca-ca 4 16.000 180.000 2.000 statistic value of parm94

nb-ca-cc-ha 4 14.500 180.000 2.000 intrpol.bsd.on C6H6

c3-c3-ce-c 6 0.000 0.000 3.000 same as X-c3-ca-X

cu-cx-c3-ca 9 1.400 0.000 3.000 same as X-c3-c3-X

nb-ca-cd-n 4 14.500 180.000 2.000 intrpol.bsd.on C6H6

ca-cx-ce-ca 6 0.000 180.000 2.000 JCC, 7, (1986), 230
